# Systematic analysis of *tup1* and *cyc8* mutants reveals distinct roles for *TUP1* and *CYC8* and offers new insight into the regulation of gene transcription by the yeast Tup1-Cyc8 complex

**DOI:** 10.1371/journal.pgen.1010876

**Published:** 2023-08-11

**Authors:** Brenda Lee, Michael Church, Karsten Hokamp, Mohamed M. Alhussain, Atif A. Bamagoos, Alastair B. Fleming

**Affiliations:** 1 Department of Microbiology, School of Genetics and Microbiology, Moyne Institute of Preventive Medicine, Trinity College Dublin, Dublin, Ireland; 2 Stowers Institute for Medical Research, Kansas City, Missouri, United States of America; 3 Department of Genetics, School of Genetics and Microbiology, Smurfit Institute, Trinity College Dublin, Dublin, Ireland; 4 Department of Biological Sciences, Faculty of Science, King Abdulaziz University, Jeddah, Saudi Arabia; The Francis Crick Institute, UNITED KINGDOM

## Abstract

The Tup1-Cyc8 complex in *Saccharomyces cerevisiae* was one of the first global co-repressors of gene transcription discovered. However, despite years of study, a full understanding of the contribution of Tup1p and Cyc8p to complex function is lacking. We examined *TUP1* and *CYC8* single and double deletion mutants and show that *CYC8* represses more genes than *TUP1*, and that there are genes subject to (i) unique repression by *TUP1* or *CYC8*, (ii) redundant repression by *TUP1* and *CYC8*, and (iii) there are genes at which de-repression in a *cyc8* mutant is dependent upon *TUP1*, and vice-versa. We also reveal that Tup1p and Cyc8p can make distinct contributions to commonly repressed genes most likely via specific interactions with different histone deacetylases. Furthermore, we show that Tup1p and Cyc8p can be found independently of each other to negatively regulate gene transcription and can persist at active genes to negatively regulate on-going transcription. Together, these data suggest that Tup1p and Cyc8p can associate with active and inactive genes to mediate distinct negative and positive regulatory roles when functioning within, and possibly out with the complex.

## Author summary

The Tup1-Cyc8 complex in the yeast, *Saccharomyces cerevisiae*, was one of the first global co-repressors of gene transcription discovered. However, despite years of study, a full understanding of this complex is lacking. We examined *TUP1* and *CYC8* single and double gene deletion mutants and show that the Tup1 and Cyc8 proteins can make distinct contributions to the regulation of Tup1-Cyc8 target genes. Furthermore, we show that Tup1p and Cyc8p can be found independently of each other to negatively regulate gene transcription and can persist at active genes to negatively regulate on-going transcription. Together, these data suggest that Tup1p and Cyc8p can associate with active and inactive genes to mediate distinct negative and positive regulatory roles when functioning within, and possibly out with the complex. This suggests the Tup1-Cyc8 complex should be considered more as a ‘regulator of transcription’ and not solely as a dedicated ‘repressor of transcription’.

## Introduction

The Tup1-Cyc8 complex in *Saccharomyces cerevisiae* was one of the first global repressors of gene transcription discovered [1]. It is known to be responsible for the repression of diverse sets of genes including those involved in the response to oxygen deprivation, DNA damage and glucose depletion [2,3].

The 1.2 MDa complex is composed of one Cyc8p and four Tup1p subunits and does not bind to DNA directly [1,4,5]. Sequence specific DNA binding proteins target the complex to the genome where evidence suggests that multiple adaptor proteins are able to fine tune its activity [1,6–8]. The Cyc8 protein (Cyc8p) has 10 copies of the 34-amino-acid tetratricopeptide repeat (TPR) motif near the N-terminus, with repeats 1–3 being the most important for interaction with Tup1p [8,9]. The Tup1 protein contains seven copies of a WD40 sequence domain (also known as β-transducin motif) at the C-terminal [10]. This domain has been shown to be required for repression of some target genes, but dispensable for repression of other target genes, such as *SUC2* [11]. Residues 1–72 of the N-terminus of Tup1p are needed for interaction with Cyc8p and for self-association, although this region is not required to bring about repression [11].

The Tup1p subunit is regarded as a functional analogue of the corepressors Groucho in *Drosophila melanogaster*, Grg in mice, and the TLE proteins in human cells [12]. Four TLE proteins are encoded in humans, TLE 1–4. They are vital for developmental processes such as sex determination, eye development, osteogenesis, and haematopoiesis [13,14]. TLE1 is important to human health as its inactivation contributes to the development of hematologic malignancies [15,16]. TLE3 has also been implicated in the proliferation of melanoma cells [17]. The homologous Tup1-Cyc8 complex in the filamentous fungi *Trichoderma reesei* and *Penicillium oxalicum* brings about repression of genes encoding for enzymes which can degrade lignocellulosic materials [18].

The general model for Tup1-Cyc8 complex activity proposes that Cyc8p acts as an adaptor protein to which the targeting proteins and Tup1p bind, while Tup1p exerts the repressive role of the complex [11]. Indeed, it has been shown that overexpression of *TUP1* is sufficient to repress transcription of Mat-a specific genes [19]. However, the *TUP1* overexpressing cells displayed a flocculant and slow-growth phenotype which suggests that *TUP1* overexpression is insufficient to repress all genes regulated by Tup1-Cyc8 [19]. Additionally, it has been shown that the complex can play a role in gene activation, albeit at fewer genes [20–24].

Multiple mechanisms of action have been proposed to describe how the Tup1-Cyc8 complex brings about gene repression [3]. The complex has been shown to associate with, and promote, hypoacetylated chromatin to repress gene transcription [25–27]. Other studies have shown it is responsible for maintaining an ordered array of nucleosomes over gene promoters to block transcription [28–33]. More recent studies suggest that it primarily blocks the activation domains of transcription factors bound at target genes to inhibit transcription [34,35].

However, the proposed mechanisms of action are not necessarily mutually exclusive. Indeed, it has been shown that at certain target genes full de-repression was only observed when multiple mechanisms of repression used by Tup1-Cyc8 were disrupted [36]. It is conceivable that different mechanisms of action are required at different genes, or that combinations of these mechanisms can determine the transcription state of genes in response to the changing environment. Regardless, this highlights that despite years of study, a complete understanding of this complex has yet to be uncovered.

Much of the current knowledge about this complex, and the genes under its control, has come from analyses of mutants deleted for either the *TUP1* or *CYC8* genes [35,37]. In support of this strategy, the current model for the activity of the complex would predict that deletion of either Tup1p or Cyc8p should have the same impact in crippling complex function. However, the common consideration of these mutants as being interchangeable for the analysis of Tup1-Cyc8 complex function ignores any differences upon transcription and cell function that deletion of these genes might have.

In this study we have compared single and double mutant strains deleted for the *TUP1* and *CYC8* genes and show that they have distinct phenotypes and transcriptomes. Almost twice as many genes were upregulated in the *cyc8* mutant compared to the *tup1* mutant. By comparing the transcription data in the single mutants to that in the double mutant we show evidence of (i) genes subject to redundant repression via *TUP1* and *CYC8*, (ii) genes which were uniquely repressed by either *TUP1* or *CYC8*, and (iii) genes at which de-repression in a *cyc8* mutant is dependent upon *TUP1*, and vice-versa. We also reveal that Tup1p and Cyc8p can make distinct contributions to commonly repressed genes. Furthermore, we show that Cyc8p and Tup1p can occupy promoters independently of each other to promote gene repression and can persist at active genes to negatively influence on-going transcription.

Together, these data suggest that Tup1p and Cyc8p have uncharacterised negative and positive roles when functioning both within and possibly out with the complex. Ultimately, the model for Tup1-Cyc8 functioning solely as a repressor of transcription is too simplistic. Instead, Tup1-Cyc8 should be considered as a more versatile regulator of transcription functioning to not only switch genes off, but also to modulate transcription of genes when they are active.

## Results

*TUP1* and *CYC8* deletion mutants have largely been used interchangeably for the analysis of Tup1-Cyc8 complex function. This was justified by the current model dictating that without either Tup1p or Cyc8p, Tup1-Cyc8 complex activity should be equally disrupted [37]. We therefore compared phenotypes and transcription in *tup1* and *cyc8* single and double deletion mutants to determine if these mutants share the same characteristics or not.

### Strains deleted for *TUP1* and *CYC8* show distinct growth, cell morphology, and flocculation phenotypes

**Fig 1:**
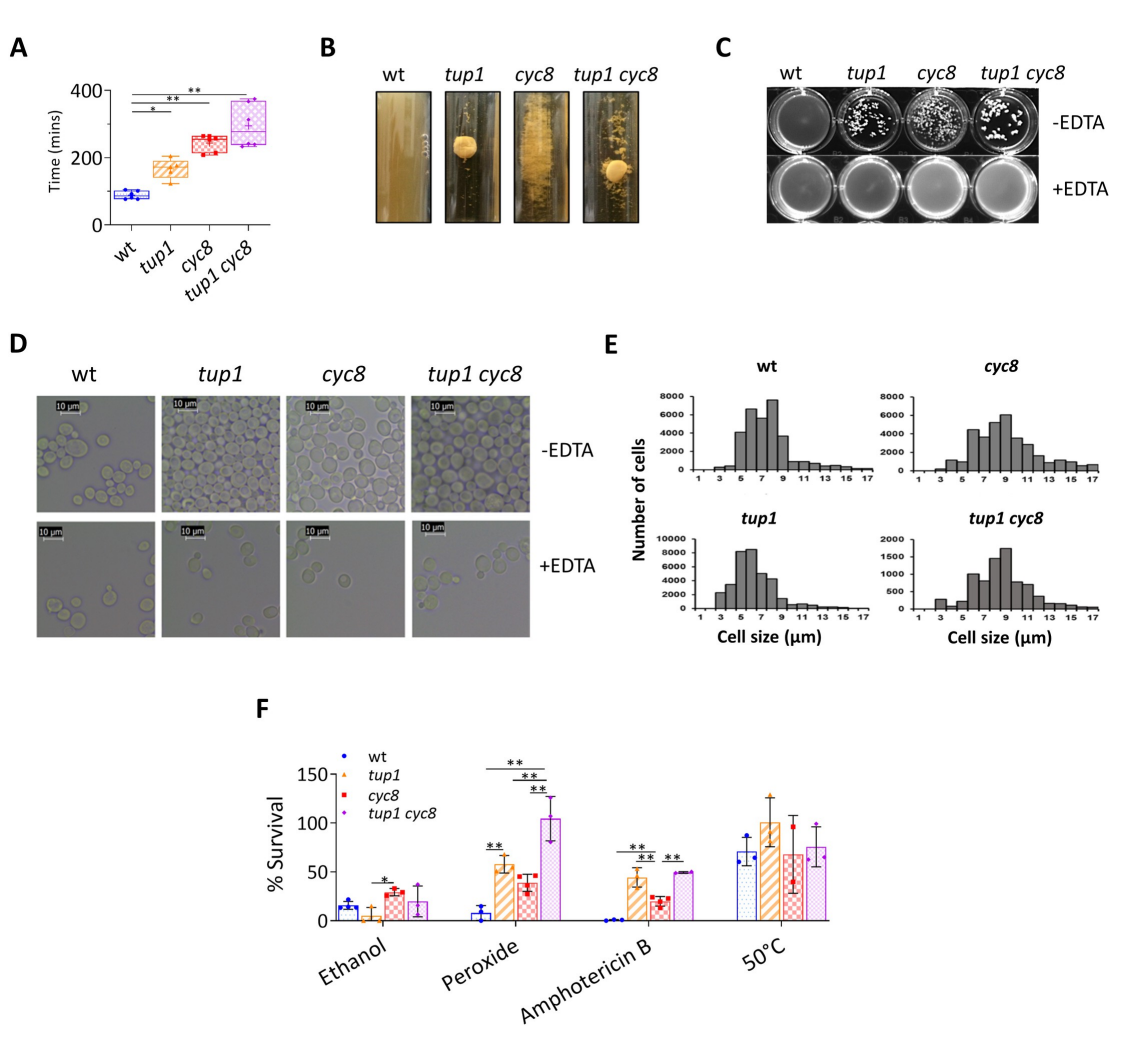
The *tup1*, *cyc8* and *tup1 cyc8* deletion mutants display different cell growth, flocculation, cell morphology and stress response characteristics. (A) Cell doubling times (min) of the strains indicated during exponential growth in YPD showing mean (+), median (line) and standard deviation from 5 biological replicates. (B) Images of cultures of the strains indicated taken after growth in YPD broth for 24 hours. (C) Exponentially growing cultures and (D) cells, were photographed in the presence (+EDTA) and absence of EDTA (-EDTA). (E) Analysis of cell size during exponential growth. Histograms were constructed representing the distribution of cell sizes in each of the indicated strains. (F) Percentage survival of exponential wt, *tup1*, *cyc8* and *tup1 cyc8* cultures incubated in the presence of the stressor indicated. Error bars represent standard deviation from 3 biological replicates (* represents a p-value of p<0.05, ** represents a p-value of p<0.005 determined by a One-way ANOVA).

Growth analysis showed that the *tup1*, *cyc8* and *tup1 cyc8* deletion mutants had progressively longer doubling times (Fig 1A). However, following growth to saturation in YPD, all strains achieved similar final cell densities.

A striking characteristic of *tup1* and *cyc8* mutant strains grown in liquid media is their strong flocculation phenotype [38,39]. Flocculation is the asexual aggregation of cells due to the expression of the *FLO* family of genes which are known to be repressed by Tup1-Cyc8 [40–42]. The *FLO* genes encode lectin-like cell wall proteins called flocculins which bind to the mannose residues within the cell walls of neighbouring cells [43]. Flocculation can be distinguished from other forms of cell aggregation by being a calcium-dependent process [44,45]. Thus, the addition of EDTA, which chelates calcium ions and disperses the flocs, can be used as a control to confirm this phenotype.

Following growth in broth, whereas the wt showed no flocculation, the *tup1* strain displayed large flocs, whilst the flocs in the *cyc8* strain were smaller and more widely dispersed throughout the liquid media (Fig 1B). The *tup1 cyc8* double deletion mutant showed an intermediate flocculation phenotype in which large flocs were visible in addition to more dispersed smaller flocs. Treatment of the *tup1*, *cyc8* and *tup1 cyc8* cultures with EDTA dispersed the cells confirming the flocculation phenotypes in each mutant (Fig 1C). Thus, deletion of *TUP1* yields the greatest visible flocculation phenotype.

When cells were visualised under the microscope, the *tup1* mutant cells were visible as large clumps of cells with little interstitial space evident (Fig 1D). The *cyc8* mutant formed smaller clumps of cells with more visible gaps between the cells. The double mutant resembled the *tup1* single mutant with large clumps of tightly packed cells being visible. These data suggest that *tup1* mutant cells might form tighter associations when flocculating compared to *cyc8* cells.

Profiling of the different EDTA-dispersed cell populations for cell size revealed that strains harbouring a *CYC8* mutation had a larger proportion of cells with larger cell sizes compared to wt or *tup1* cells (Fig 1E).

### Strains deleted for *CYC8* and *TUP1* have different responses to stressors

Flocculation is a stress response in which cells on the inside of a floc are shielded from chemical stressors which cannot easily infiltrate the tightly packed cells [46]. We therefore investigated the ability of each strain to tolerate a variety of stressors (Fig 1F).

When cells were exposed to ethanol, which can lead to loss of membrane integrity, wt survival was reduced to 20%. However, survival of the *tup1* mutant was 2-fold less than wt, survival of the *cyc8* mutant was higher than wt, and survival in the double mutant was similar to wt. Thus, the *cyc8* mutant showed the greatest resistance to ethanol, despite this strain showing the weakest visible flocculation phenotype. Conversely, the highly flocculant *tup1* mutant was more sensitive to ethanol than wt.

When cells were exposed to H_2_O_2_, which can cause oxidative damage, wt survival was reduced to 10% whereas all the mutants showed significantly increased survival. The *tup1* mutant showed more survival than the *cyc8* mutant, whilst survival was the greatest in the double mutant. Thus, resistance to H_2_O_2_ was greatest in the *tup1 cyc8* double mutant which showed an intermediate flocculation phenotype.

Exposure of cells to amphotericin B, an antifungal drug which targets the cell membrane, caused almost 100% cell death in wt. However, all the mutants showed significantly increased survival levels with survival in the *tup1* mutant being >2-fold higher than that in the *cyc8* mutant. The double mutant showed survival levels similar to the *tup1* mutant.

The ability of the strains to tolerate high temperatures in liquid culture was included as a control, as temperature should affect all cells equally regardless of the extent of flocculation [46]. As expected, following exposure of cells to high temperature (50°C), no significant difference in survival in any of the strains could be detected.

Thus, the mutants showed differences in their resistance to chemical stressors that were not always dependent upon their flocculant phenotypes.

### Strains deleted for *TUP1* de-repress *FLO1* gene transcription to the greatest extent

**Fig 2:**
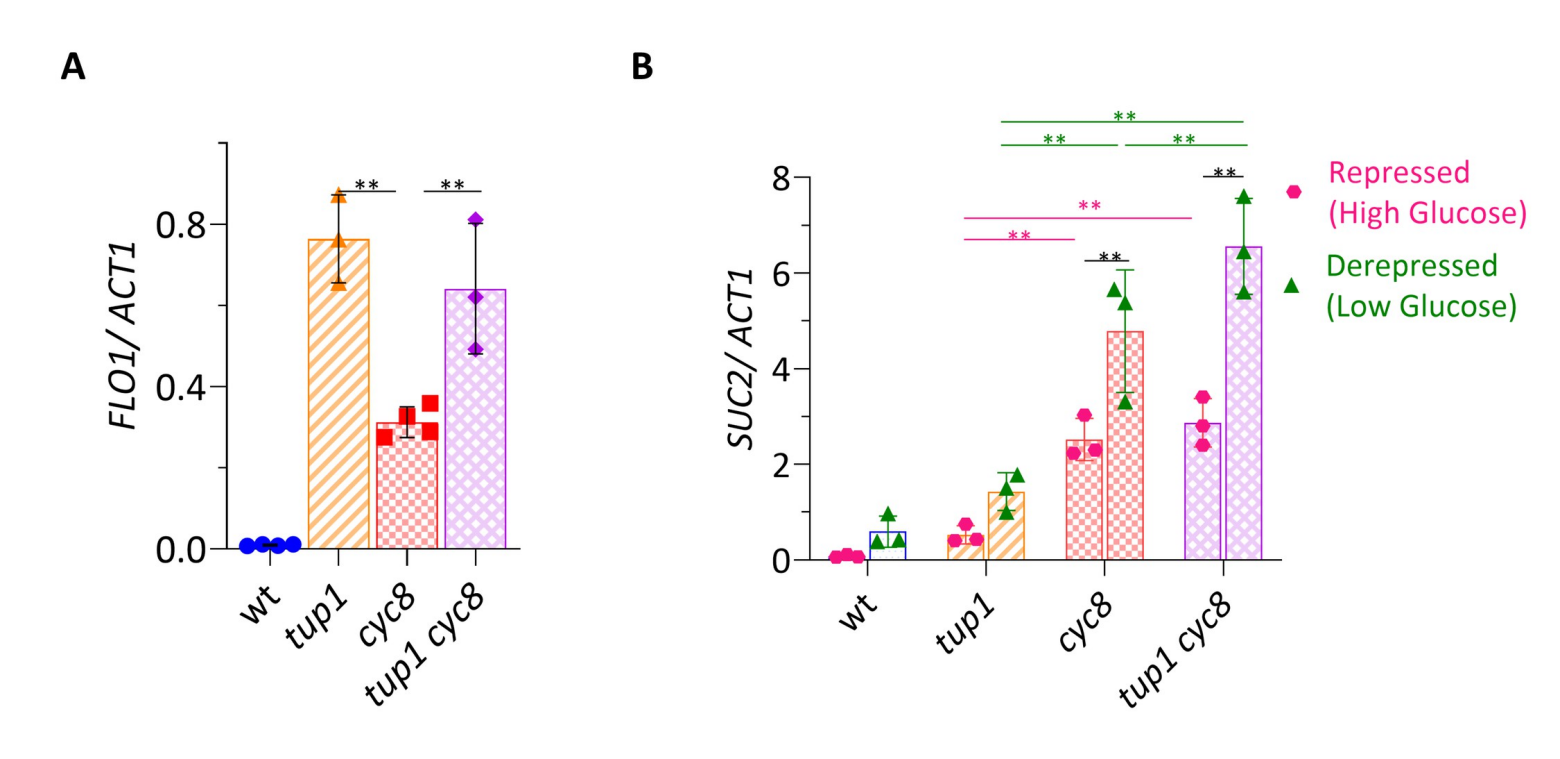
*tup1* and *cyc8* mutants de-repress the *FLO1* and *SUC2* genes to different extents. (A) *FLO1* transcript levels measured using RT-qPCR. The fold change in *FLO1* de-repression in *tup1*, *cyc8* and *tup1 cyc8* relative to wt was 78-, 31- and 66-fold, respectively. (B) *SUC2* transcript levels measured following growth on high (Repressed) and low (De-repressed) glucose levels. In all graphs, values were normalised to *ACT1* mRNA and error bars reflect standard deviation from 3–4 biological replicates (** represents a p-value of p<0.005 determined by a One-way ANOVA analysis).

The most striking phenotype displayed by the mutant strains was the flocculation phenotype which is mediated by expression of the *FLO* family of genes [42]. We therefore measured *FLO* gene transcription in each mutant and wt (Figs 2A and S1).

The mRNA levels of the *FLO* genes were low in wt, consistent with Tup1-Cyc8 dependent repression of transcription, and correlating with the lack of flocculation in the parent strain. Consistent with *FLO1* being the dominant flocculation gene, *FLO1* mRNA levels were the highest of the *FLO* genes tested in each of the mutants (Figs 2A and S1). *FLO1* mRNA levels were greater in the *tup1* mutant than in the *cyc8* mutant. Interestingly, the fact that *FLO1* transcription in the *cyc8* single mutant was further de-repressed when *TUP1* was additionally deleted (Fig 2A, compare *cyc8* and *tup1 cyc8*) suggests that *TUP1* can exert a repressive effect upon *FLO1* in the absence of *CYC8*. Thus, the extent of *FLO1* de-repression correlates with the flocculation phenotypes of each strain and *TUP1* makes the major contribution to *FLO1* repression.

### Strains deleted for *CYC8* de-repress *SUC2* gene transcription to the greatest extent

Another well-characterised gene subject to Tup1-Cyc8 dependent repression is the *SUC2* gene [47]. *SUC2* encodes invertase which hydrolyses sucrose to yield glucose and fructose. The gene is repressed in the presence of high levels of glucose and is induced under conditions of low glucose [48,49]. We therefore analysed *SUC2* transcription in the *cyc8* and *tup1* single and double mutants under conditions of high and low glucose (Fig 2B).

Consistent with published data, *SUC2* mRNA in wt was barely detectable under conditions of high glucose (repressed), whilst the gene was significantly de-repressed under conditions of low glucose (de-repressed) (Fig 2B, wt) [49].

In the *tup1* mutant, the level of *SUC2* mRNA in the repressed (high glucose) condition was similar to the *SUC2* mRNA present in the wt strain under the de-repressed (low glucose) condition (Fig 2B, *tup1*). This is consistent with the loss of glucose repression of *SUC2* in the *tup1* mutant due to disruption of the Tup1-Cyc8 complex [48]. However, *SUC2* mRNA levels were even greater when the *tup1* mutant was grown under low glucose conditions.

In both the *cyc8* and the *tup1 cyc8* double mutant strains grown on high glucose, *SUC2* mRNA levels were higher than levels found in both the repressed and de-repressed *tup1* cells (Fig 2B). *SUC2* mRNA was also further elevated in the *cyc8* and the *tup1 cyc8* double mutant cells when grown under low glucose conditions.

Together, these data show that in the wt strain under conditions of low glucose, *TUP1* and *CYC8* still exert a repressive effect upon *SUC2* transcription. The fact that transcription in the *tup1* single mutant is further de-repressed in either the repressed or de-repressed conditions when *CYC8* is additionally deleted (Fig 2B, compare *tup1* and *tup1 cyc8*) suggests that the lower levels of *SUC2* mRNA in the *tup1* mutant under either glucose condition is dependent upon *CYC8*. Finally, these data show that even in the absence of both Tup1p and Cyc8p, *SUC2* is not fully de-repressed when glucose is present.

Thus, *CYC8* makes the major contribution to *SUC2* repression and can exert a repressive effect upon *SUC2* transcription in the absence of *TUP1*. Furthermore, *TUP1* and *CYC8* continue to exert a repressive effect upon *SUC2* transcription during the de-repressing conditions associated with a low-glucose environment.

### Different numbers of genes are upregulated in *cyc8* and *tup1* deletion mutants

We next examined global transcription in the *tup1*, *cyc8* and *tup1 cyc8* mutants to determine if there were more widespread differences in gene transcription in strains deleted for *TUP1* and/or *CYC8*.

**Fig 3:**
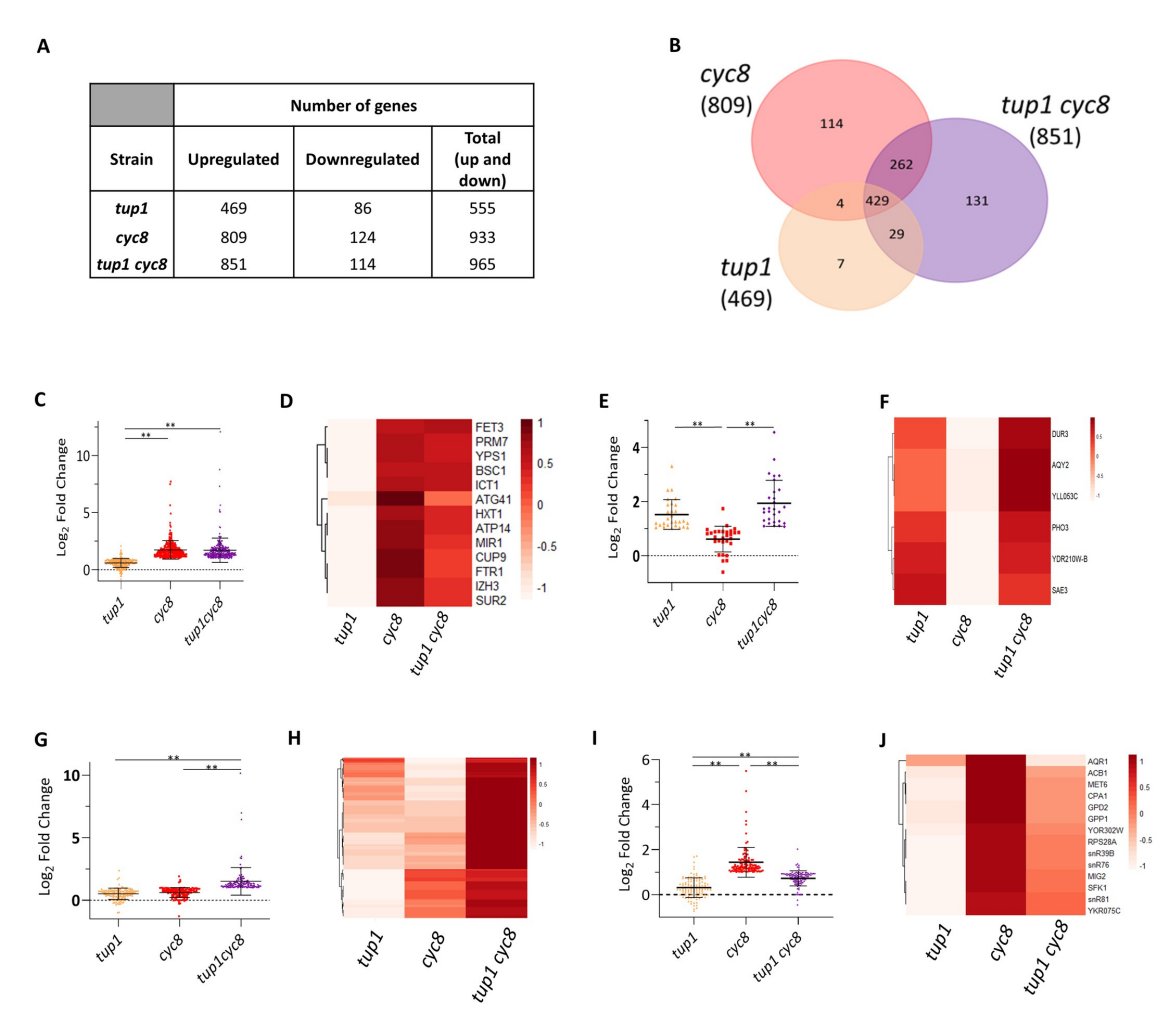
Comparison of genes up and downregulated in *tup1*, *cyc8*, and *tup1 cyc8* mutants compared to wt. (A) Table showing the number of genes at least two-fold up- and downregulated in each mutant compared to wt (|log2 fold-change| ≥ 1, adjusted p-value p ≤ 0.01). (B) Venn diagram of all genes that are at least two-fold upregulated in *tup1*, *cyc8* and *tup1 cyc8*, compared to wt. (C) Scatterplot showing the log_2_ fold change values of the 262 genes that were upregulated only in the *cyc8* and *tup1 cyc8* mutant strains. (D) Cluster heatmap for the 13 genes designated as uniquely repressed via *CYC8*. (E) Scatterplot showing the log_2_ fold change values of the 29 genes that were upregulated only in the *tup1* and *tup1 cyc8* mutants. (F) Cluster heatmap for the 6 genes identified as being subject to unique repression via *TUP1*. (G) Scatterplot showing the log_2_ fold change values of the 131 genes upregulated only in the *tup1 cyc8* double mutant. (H) Cluster heatmap for the 131 genes subject to potential redundant *TUP1* and *CYC8* repression. (I) Scatterplot showing the log_2_ fold change values of the 114 genes upregulated only in the *cyc8* mutant compared to wt. (J) Cluster heatmap for the 14 genes which showed no, or minimal upregulation in the absence of *TUP1*, but were upregulated in the absence of *CYC8*. In all graphs, error bars reflect standard deviation (* represents a p-value of p<0.05, ** represents a p-value of p<0.005 determined by ANOVA analysis). Each heatmap displays Z-scores for each gene; each row represents a gene, and each column represents a deletion mutant. The colour scale indicates the standard deviations above or below the mean fold change for each gene compared to wt.

Consistent with Tup1-Cyc8 having been best characterised as a co-repressor of gene transcription, 469 genes were upregulated in the *tup1* mutant, 809 genes were upregulated in the *cyc8* mutant, and 851 genes were upregulated the *tup1 cyc8* double mutant (Fig 3A) [2]. Conversely, only 86, 124 and 114 genes were downregulated more than two-fold in the *tup1*, *cyc8* and *tup1 cyc8* mutants, respectively. We therefore focussed our analysis on the genes that were upregulated in the *tup1*, *cyc8* and *tup1 cyc8* mutant strains compared to wt, where *TUP1* and *CYC8* could be inferred to be playing a role in gene repression.

According to the current model for Tup1-Cyc8 complex activity, it would be predicted that if Tup1p and Cyc8p functioned solely within the Tup1-Cyc8 complex, the same genes would be upregulated in each of the single and double mutants. However, there were 7, 114 and 131 genes exclusively upregulated in the *tup1*, *cyc8* and *tup1 cyc8* mutants, respectively (Fig 3B), suggesting unique cohorts of *TUP1* and *CYC8* repressed genes.

### Genes showing independent repression by *CYC8* and *TUP1*

The data showing 262 genes upregulated only in the *cyc8* and *tup1 cyc8* mutants compared to wt suggests that these genes are subject to unique repression by *CYC8* (Fig 3B). Although a scatter plot confirms that the average upregulation of transcription of these genes in the *cyc8* and *tup1 cyc8* mutants was greater than transcription in the *tup1* mutant (Fig 3C), to identify those genes solely repressed via *CYC8* more accurately we set three, more stringent, parameters. Firstly, we proposed that the genes must show minimal transcription in the *tup1* mutant compared to wt (cut off = Log_2_ fold-change (FC) ≤0.3). Secondly, upregulation of these genes should not be higher in the *tup1 cyc8* double mutant compared to the *cyc8* mutant. Thirdly, we excluded genes showing very low transcription levels in the mutants (cut off average transcripts per million (TPM) ≤60). This analysis uncovered 13 genes which we propose are uniquely repressed via *CYC8* in wt (Figs 3D, S2A and S2B). A similar analysis of the 29 genes upregulated only in the *tup1* and *tup1 cyc8* mutants (Fig 3B and 3E) revealed 6 genes uniquely repressed via *TUP1* (Figs 3F, S2C and S2D).

### Genes subject to redundant repression by *CYC8* and *TUP1*

We next investigated the 131 genes that were upregulated at least two-fold only in the *tup1 cyc8* double mutant (Fig 3B, 3G and 3H). This result indicates that each subunit can compensate for the absence of the other to bring about repression at these genes. Full gene de-repression is only achieved when both subunits are deleted. An example of a gene subject to potential redundant repression via *TUP1* and *CYC8* was *FIT2* (S2E Fig).

### Evidence of *CYC8*-repressed genes subject to positive regulation via *TUP1*

We next examined the genes which were de-repressed in either or both *tup1* and *cyc8* single mutants, but which were not de-repressed in the double mutant (Fig 3B). For example, there were 114 genes significantly upregulated only in the *cyc8* mutant (Fig 3B and 3I). This profile suggests that these genes were subject to unique *CYC8*-dependent repression but were *TUP1*-dependent for transcription in the absence of *CYC8*. To increase the stringency for this gene cohort, genes showing very low levels of transcription (average TPM of <60) in each of the mutants were discarded, and genes showing any change in transcription in the *tup1* mutant were also excluded. This revealed a cohort of 14 *CYC8* repressed genes at which *TUP1* was required for their transcription in the absence of *CYC8* (Figs 3J and S2F).

### Distinct levels of gene de-repression occur in *tup1* and *cyc8* mutants

We next examined the transcription levels of all the genes upregulated in the *tup1*, *cyc8* and *tup1 cyc8* mutants to determine if there were any general differences in the levels of gene de-repression in any of these strains which might indicate distinct contributions to gene repression by *TUP1* or *CYC8*.

**Fig 4:**
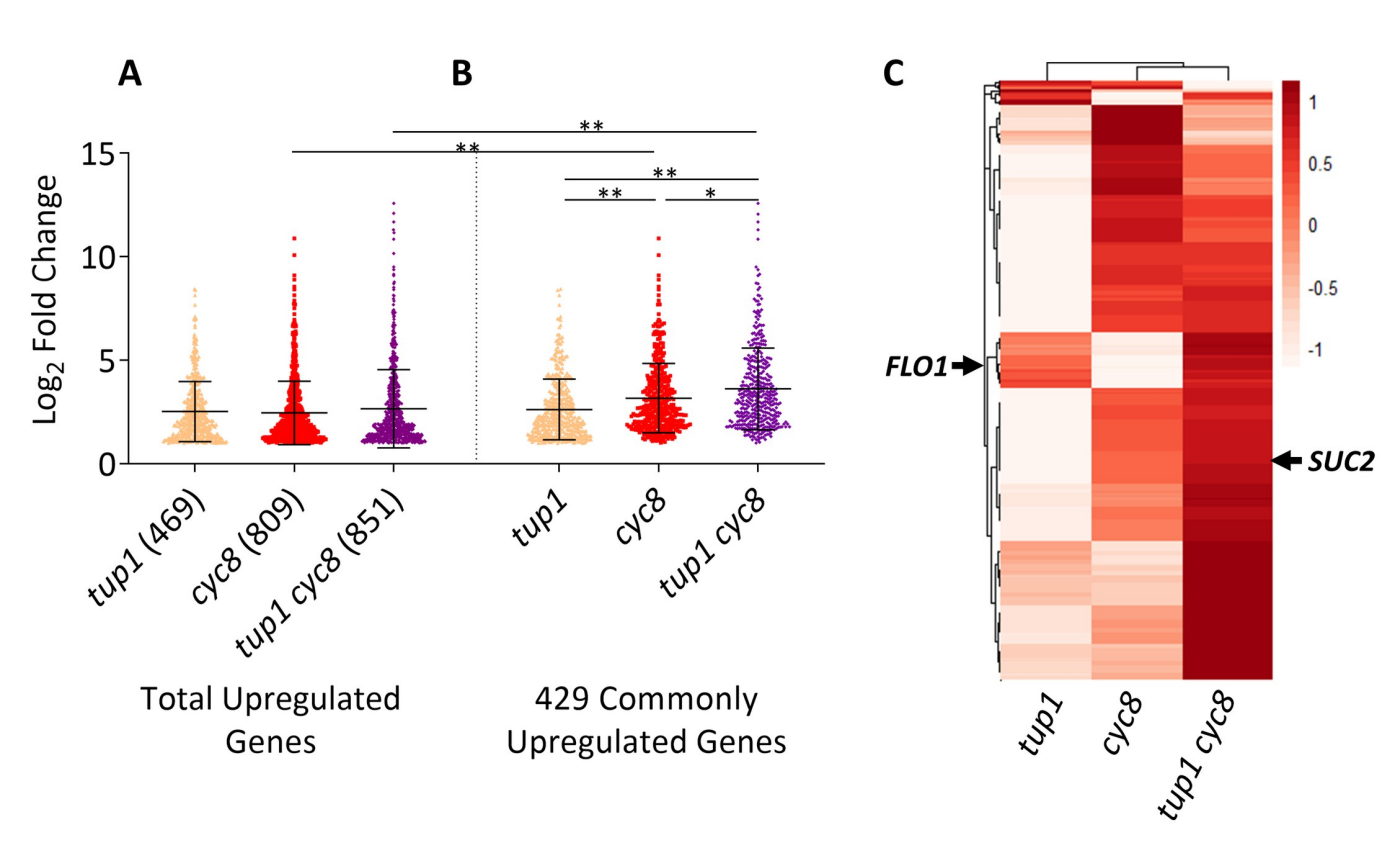
Commonly upregulated genes are upregulated by different amounts in each mutant strain. Scatterplots showing the log_2_ fold change values of (A) all genes at least two-fold upregulated in each of the deletion strains compared to wt, and (B) the 429 genes commonly upregulated in all three deletion mutants compared to wt. In each case, log_2_≥1, adjusted p-value of p≤0.01, mean, and standard deviation are shown (* represents a p-value of p≤0.05, ** represents a p value of p≤0.005 as determined by ANOVA analysis). (C) Cluster heatmap for the 429 genes at least two-fold upregulated in all three deletion strains compared to wt. The heatmap displays Z-scores for each gene; each row represents a gene, and each column represents a deletion mutant. The colour scale indicates the standard deviations above or below the mean fold change for each gene compared to wt.

Comparison of the levels of de-repression of the total number of genes de-repressed in the *cyc8* (809), *tup1* (469) and *tup1 cyc8* (851) strains revealed no difference in their average levels of de-repression (Fig 4A). However, examination of the 429 genes commonly upregulated in the *tup1*, *cyc8* and *tup1 cyc8* mutants revealed average upregulation was the least in *tup1*, was higher in *cyc8*, and was highest in *tup1 cyc8* (Fig 4B). Furthermore, the average upregulation of these commonly repressed genes in the *cyc8* and *tup1 cyc8* mutants was significantly greater than the average upregulation of the total number of genes de-repressed in these strains (compare Fig 4B to 4A). This indicates that the set of 429 commonly repressed genes represents a core set of genes that are subject to (i) robust repression by *TUP1* and *CYC8* and (ii), distinct levels of repression by *TUP1* and *CYC8*, with *CYC8*, on average, making the greater contribution to their repression than *TUP1*.

### There are distinct cohorts of genes commonly repressed by *TUP1* and *CYC8*

Visualisation of the relative levels of transcription of the 429 commonly de-repressed genes in the different mutants via a heat map confirmed that most genes had the highest de-repression in the *tup1 cyc8* mutant (Fig 4C). However, 110 genes had the highest de-repression in the *cyc8* mutant and the lowest de-repression in the *tup1* mutant (Fig 4C). An example of a gene showing this transcription profile was *SUC2* (see Fig 2B). Additionally, there was a cohort of 40 genes which showed greater de-repression in the *tup1* mutant compared to the *cyc8* mutant, of which, *FLO1* was an example (Fig 4C, see Fig 2A). There were also genes, such as *RNR3*, which were equally upregulated in each mutant (S3 Fig) [50].

### The commonly repressed genes subject to differential *TUP1* and *CYC8* repression are enriched within distinct sub-telomeric regions

In a large proportion of the 429 genes commonly upregulated in the *tup1*, *cyc8* and *tup1 cyc8* mutants, transcription was de-repressed the most in a *cyc8* mutant compared to *tup1* (see Fig 4B and 4C). An example of this cohort of genes was *SUC2*. Conversely, *FLO1* was representative of the smaller subset of the 429 commonly de-repressed genes which were most de-repressed in the *tup1* mutant. We therefore investigated these two cohorts of commonly repressed genes (*SUC2*-type and *FLO1*-type genes) to determine if there were any unique characteristics that might explain why their repression was most dependent on either *CYC8* or *TUP1*.

**Fig 5:**
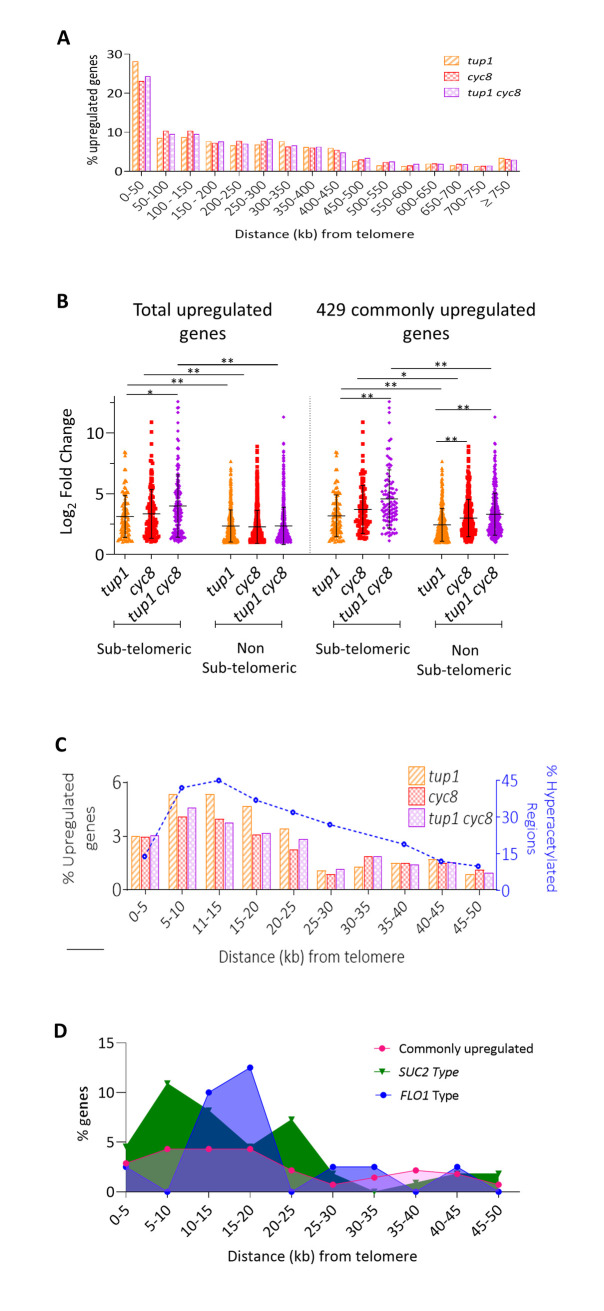
Distribution of *FLO1*- and *SUC2*-type genes across the genome. (A) Column graph showing the total upregulated genes percentage of location across the chromosomes. Distance is in kilobase (kb) pairs from each telomere grouped into 50 kb regions. (B) Scatterplot of the log_2_ fold changes of the total upregulated genes and the 429 commonly upregulated genes separated into genes located within the sub-telomeric regions of the chromosomes (<25 kb from the telomeres) and the genes located throughout the rest of the chromosomes (Non sub-telomeric). Mean and standard deviation are shown, * represents a p-value of p≤0.05, ** represents a p-value of p≤0.005 as determined by ANOVA analysis. (C) Graph showing distribution of genes significantly upregulated in each mutant compared to wt (% of total upregulated genes) located in the first 50 kb from each telomere grouped into 5 kb regions (columns, left-hand Y axis), and the Hda1-affected sub-telomeric (HAST) domain (dashed line, right-hand Y axis). Distance is in kilobase pairs from each telomere. Right-hand Y axis indicates regions of hyperacetylation (HAST domain) in an *hda1* deletion mutant; adapted from Robyr *et al*., 2002 [62]. (D) Distribution of the 429 commonly upregulated genes over the first 50 kb regions from the telomeres divided into 5 kb regions and separated into *FLO1*-type (blue), *SUC2*-type (green), and the remaining commonly upregulated genes (pink). The percentage of genes in each group was calculated.

Gene-ontology analysis did not reveal any distinction between these two cohorts of commonly repressed gene. Consistent with previous studies, the majority of the total *TUP1* and *CYC8* repressed genes were enriched near the ends of chromosomes (Fig 5A), and that genes within sub-telomeric regions were subject to the most robust repression via *CYC8* and *TUP1* (Fig 5B) [27]. Further analysis revealed that the distribution of total *TUP1* and *CYC8* repressed genes correlated well with the Hda1-affected sub-telomeric (HAST) domains located between 5 and 40 kb of a telomere (Fig 5C) [51]. Interestingly, within the HAST domain, the *FLO1*-type genes were tightly enriched within the 15–20 kb sub-telomeric region, whilst the *SUC2*-type genes were enriched either side of this region (Figs 5D and S4).

Analysis of the basal level of transcription of the *SUC2*- and *FLO1*-type genes in wt revealed that, overall, the two sets of genes showed no difference in their low TPM values in wt (S5 Fig). This suggests both gene cohorts were equally robustly repressed in wt.

Previous studies had shown that *FLO1* and *SUC2* were subject to long-range antagonistic chromatin remodelling by Tup1-Cyc8 and Swi-Snf in their extensive gene transcription-free upstream regions [28–30]. We therefore examined if the *FLO1*- and *SUC2*-type genes had different lengths of upstream or downstream gene-free regions which might influence their regulation (S6A and S6B Fig). The results showed no significant difference in the average length of up and downstream intergenic regions, or open reading frame (ORF) lengths, between the two sets of genes. However, there was a positive correlation between the length of the upstream intergenic region and the levels of gene de-repression for the *FLO1*-type genes in the *tup1 cyc8* double mutant, and for the *SUC2*-type genes in the *cyc8* mutant (S6C and S6D Fig). Thus, repression of both sets of genes are similarly influenced by the extent of their gene-free upstream regions.

We next analysed transcription factor (TF) consensus sequences in the promoter regions of the two gene cohorts (S1 Table). This showed that the average number of motifs found upstream of the two sets of genes was very similar, with 111.6 for the *FLO1*-type genes, and 111.25 for *SUC2*-type genes. However, at *SUC2*-like genes, there was an enrichment of binding sites for Nrg1p, Msn2p and Hap1p, whereas binding sites for Yap1p, Hac1p and Gcn4p were enriched at the *FLO1*-type genes.

Thus, the two cohorts of commonly repressed genes differ in their distribution within the HAST domains of the sub-telomeric regions and are associated with different transcription factor binding sites.

### Cyc8p can occupy the *SUC2* promoter in the absence of Tup1p

**Fig 6:**
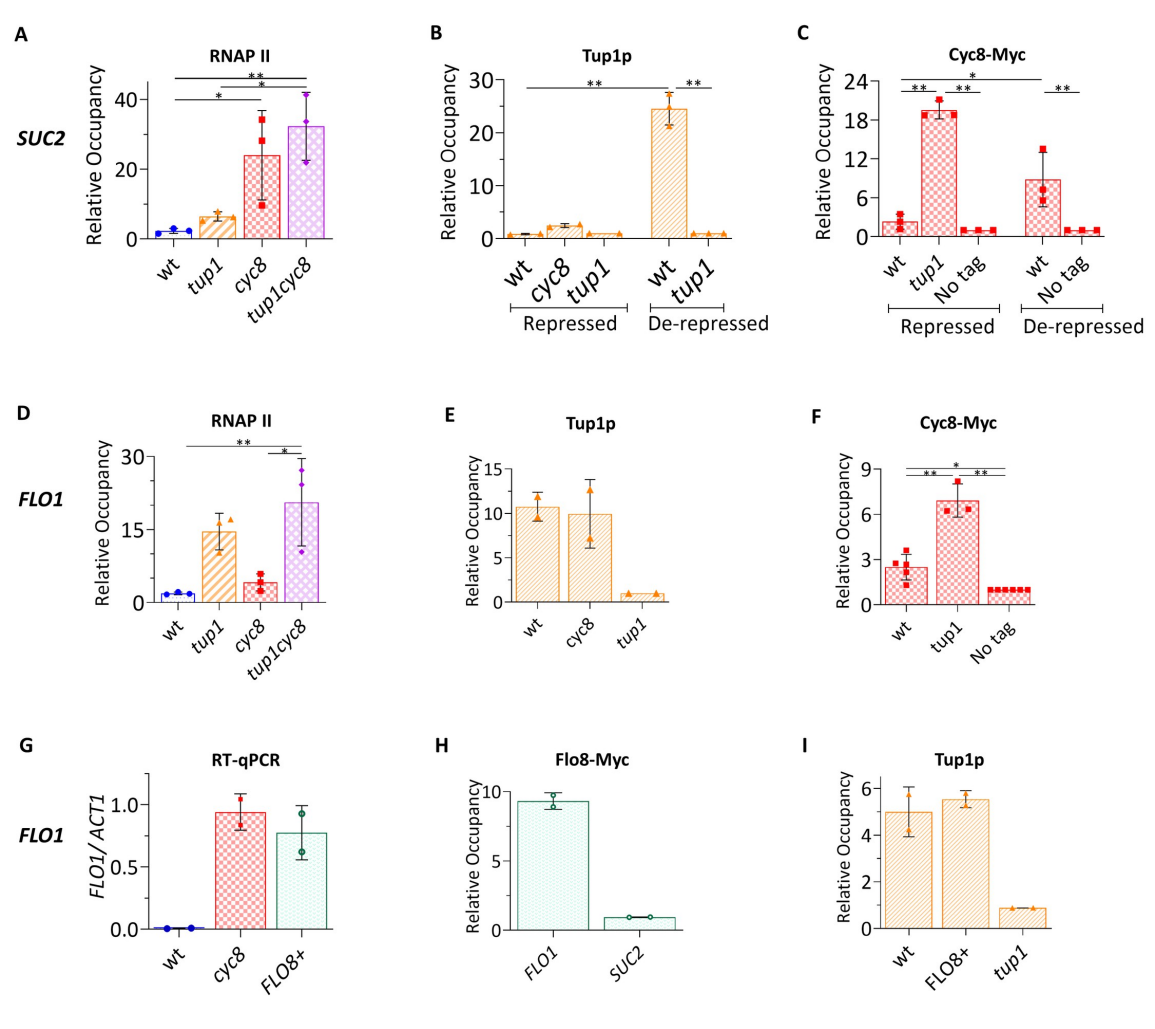
Tup1p and Cyc8p occupancy at the *SUC2* and *FLO1* promoters. (A) RNA polymerase II (RNAP II) occupancy at the *SUC2* open reading frame (ORF) in wt, *tup1*, *cyc8* and *tup1 cyc8* in glucose grown cells. RNAP II signals (IP/IN) were normalised to an internal negative control region (IP/IN at *Tel-VI*) (n = 3). (B) ChIP analysis of Tup1p occupancy at the *SUC2* promoter region in repressed (high glucose) and de-repressed (low glucose) conditions in the strains indicated. Tup1p IP/IN values were normalised to an internal negative control region (IP/IN at *Tel-VI*) and plotted relative to the *tup1* mutant (n = 3). (C) ChIP analysis of Cyc8-Myc occupancy at the *SUC2* promoter region in the strains indicated. Cyc8-Myc IP/IN values were normalised to an internal negative control region (IP/IN at *Tel-VI*) and plotted relative to an untagged (No tag) strain (n = 3). (D) RNA polymerase II (RNAP II) occupancy at the *FLO1* ORF in glucose grown cells. ChIP was carried out as described in (A). (E) Tup1p occupancy at the *FLO1* promoter. ChIP was carried out as described in B. (F) Cyc8-Myc occupancy at the *FLO1* promoter. ChIP was carried out as described in C. (G) *FLO1* transcript levels measured relative to *ACT1* mRNA levels using RT-qPCR in wt, *cyc8*, and a strain with a functional *FLO8* ORF (*FLO8*^+^) (n = 2). (H) Flo8-Myc occupancy at the *FLO1* and *SUC2* promoter. Flo8-Myc IP/IN values at the promoter regions were normalised to an internal negative control region (IP/IN at *Int-V*) as described in S8E Fig and F (n = 2). (I) Tup1p occupancy at the *FLO1* promoter in wt, *FLO8*^+^ and *tup1*. ChIP was carried out as described in (B) (n = 2). (D-I) All cells were grown in YPD (glucose at 2%). For all plots, mean and standard deviation are shown from 2–5 biological replicates; asterisks represent a p-value of * = p≤0.05, ** = p≤0.005 obtained from One-Way ANOVA analysis. Examples to illustrate normalisation steps used for RNAP II, Cyc8-Myc and Tup1p ChIP are shown in S11 Fig.

In the majority of the 429 genes commonly upregulated in the *tup1*, *cyc8* and *tup1 cyc8* mutants, gene transcription was de-repressed the least in a *tup1* mutant compared to the de-repression in the *cyc8* and *tup1 cyc8* double mutants (see Fig 4B and 4C). This suggests that there was a *CYC8*-dependent repressive effect upon transcription of these genes in the absence of *TUP1*. To test whether the repressive role of *CYC8* in the absence of *TUP1* might be direct or not, we performed chromatin immunoprecipitation (ChIP) analysis of Cyc8p and Tup1p occupancy using *SUC2* as an example of this cohort of genes in the glucose-grown strains (Fig 6). We first performed ChIP analysis of RNA polymerase II (RNAP II) at the *SUC2* gene to confirm the RNA-seq and RT-qPCR data (Fig 6A). Consistent with the transcription data, we detected low levels of RNAP II at the *SUC2* ORF in wt, compared to high levels in the *cyc8* and *tup1 cyc8* mutant (compare Figs 6A and 2B, repressed).

Surprisingly, we could not detect significant enrichment of Tup1p or Cyc8p at the repressed *SUC2* promoter in the wt strain at the previously reported site of Tup1-Cyc8 occupancy (Fig 6B and 6C; wt, repressed) [52,53]. We suggest this discrepancy is due to differences in the antibodies and ChIP signal normalisation strategies used between labs. However, supported by the abundance of literature detailing Tup1-Cyc8 repression of *SUC2*, we propose that Tup1p and Cyc8p are present at the repressed *SUC2* promoter, but are not detectable by ChIP using our conditions [3,37,47]. Consistent with the model for Tup1-Cyc8 function, we also could not detect significant occupancy of Tup1p at *SUC2* in the *cyc8* mutant (Fig 6B; *cyc8*, repressed). However, we could detect significant enrichment of Cyc8p at the partially de-repressed *SUC2* promoter in the *tup1* mutant (Fig 6C; *tup1*, repressed). Thus, Cyc8p was present at the partially de-repressed *SUC2* promoter in the absence of Tup1p where it could contribute directly to negatively influencing *SUC2* transcription.

### Tup1p is detectable at *FLO1* in the absence of Cyc8p

We next looked at RNAP II, Tup1p and Cyc8p occupancy at the *FLO1* gene which was representative of the smaller subset of the 429 commonly repressed genes (Fig 6D–6F). De-repression at these genes was greater in the *tup1* and *tup1 cyc8* double mutant compared to de-repression in the *cyc8* mutant, suggesting a *TUP1*-dependent repressive effect upon transcription of these genes in the absence of *CYC8* (see Fig 4C). Firstly, the RNAP II ChIP results were consistent with the mRNA levels detected in the strains whereby there were low RNAP II levels in wt, and high RNAP II levels in the *tup1* and *tup1 cyc8* mutants (compare Figs 6D and 2A). Consistent with Tup1-Cyc8 mediated repression of *FLO1*, Tup1p and Cyc8p occupancy could be detected at the wt *FLO1* promoter when the gene was off (Fig 6E and 6F, wt) [27,54]. Similar to what was seen at *SUC2*, Cyc8p was also detected at significant levels at *FLO1* in the *tup1* mutant (Fig 6F, *tup1*). Most interestingly, Tup1p could be detected at the *FLO1* promoter in the absence of Cyc8p (Fig 6E, *cyc8*).

Therefore, ChIP analysis confirmed that Tup1p and Cyc8p could be detected at the repressed *FLO1* promoter. The data also revealed that Cyc8p could be detected at high levels at *FLO1* and *SUC2* in the absence of Tup1p, and that Tup1p could be detected at the *FLO1* promoter in the absence of Cyc8p. Thus, Cyc8p could directly contribute to repression of *SUC2* transcription independent of Tup1p, and Tup1p could directly contribute to *FLO1* repression in the absence of Cyc8p.

### Tup1p and Cyc8p are present at the active *SUC2* and *FLO1* genes

Previous work had suggested that the Tup1-Cyc8 complex remains at some genes, including *SUC2*, following gene activation [21,23,52,53]. We therefore analysed Tup1p and Cyc8p occupancy at *SUC2* following its activation in response to low glucose conditions (Fig 6B and 6C; wt, de-repressed). Consistent with previous data, we confirmed that Tup1p and Cyc8p could be detected at the *SUC2* gene following its induction.

In most laboratory strains, including the ones used in this study, flocculation is an undesirable phenotype and has been attenuated by a nonsense mutation in the *FLO8* gene which encodes an activator of the *FLO* genes [55,56]. Thus, most studies to investigate *FLO1* transcription employ *tup1* or *cyc8* mutants in which repression of *FLO1* is abolished. To examine Tup1p and Cyc8p occupancy at the active *FLO1* gene under non-mutant conditions we restored the wt *FLO8* gene at its genomic locus prior to performing ChIP (S7, S8A and S8B Figs). Following restoration of a functional *FLO8* gene, the *FLO8+* strain exhibited a flocculant phenotype and showed *FLO1* mRNA levels similar to those in the *cyc8* mutant (Fig 6G). Subsequent tagging of the restored *FLO8* gene allowed us to confirm that Flo8p was expressed, and ChIP analysis revealed that Flo8p could specifically occupy the *FLO1* promoter (Figs 6H, S8B, S8E and S8F). Together, this suggests that when Flo8p is expressed under the control of its native promoter it can bind the *FLO1* promoter to activate *FLO1* transcription.

ChIP analysis for Tup1p occupancy at the *FLO1* promoter in the *FLO8+* strain, where *FLO1* is being transcribed at levels similar to that in the *cyc8* mutant, revealed that Tup1p was present (Fig 6I). Furthermore, the levels of Tup1p detected at the active *FLO1* gene in the *FLO8+* strain were similar to those seen in the wt strain when *FLO1* transcription was repressed. This suggests that Tup1p, most likely in the context of the Tup1-Cyc8 complex, can persist at the *FLO1* gene when it is being actively transcribed.

### Tup1p and Cyc8p have unique and shared sites of occupancy across the genome

**Fig 7:**
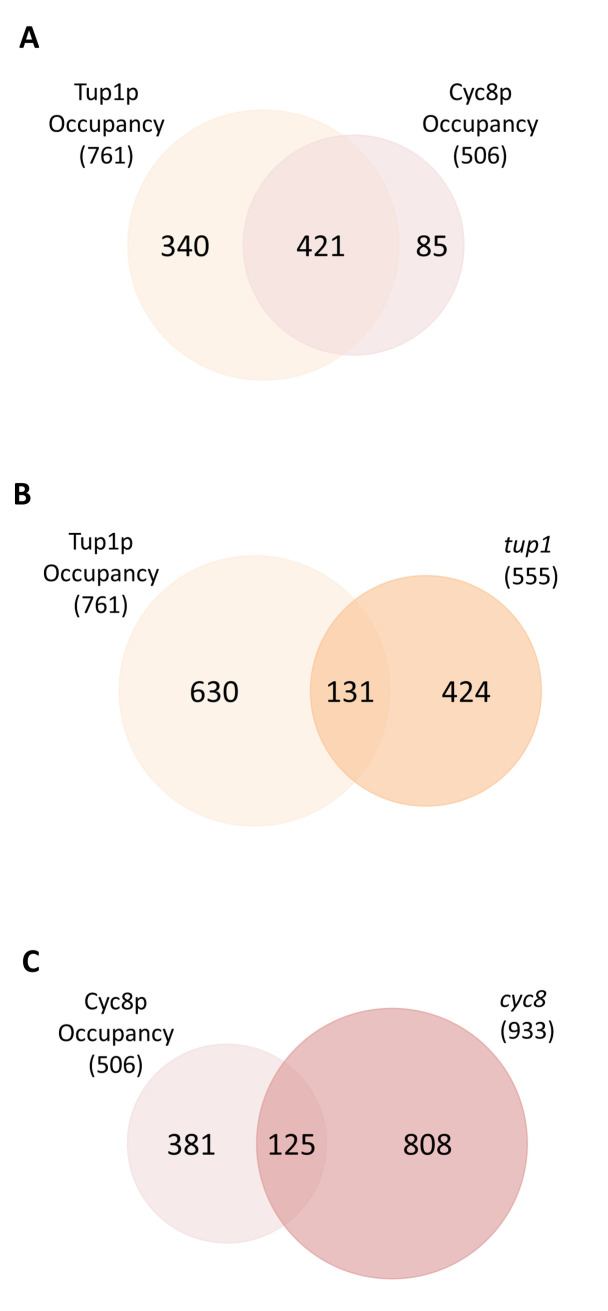
Global Tup1p and Cyc8p occupancy. (A) Venn diagram showing the overlap between global Tup1p and Cyc8p occupancy at annotated genes. Tup1p and Cyc8p occupancy data were from ChIP-Exo data retrieved from Rossi *et al*., 2021 [57]. (B) Venn diagram showing the overlap between global Tup1p occupancy and genes differentially transcribed in the *tup1* deletion mutant (*tup1*). (C) Venn diagram showing the overlap between global Cyc8p occupancy and genes differentially transcribed in the *cyc8* deletion mutant (*cyc8*).

Finally, we examined the global occupancy of Tup1p and Cyc8p using previously published data obtained via ChIP-Exo (Fig 7) [57]. The data showed there were more sites of occupancy detected for Tup1p (761) than for Cyc8p (506) and that, although there was a significant overlap in the Tup1p and Cyc8p sites of occupancy (421 genes), Tup1p and Cyc8p could be detected at a significant number of sites independently from each other (Fig 7A). Indeed, Tup1p could be found at 340 unique sites whilst Cyc8p was located at 85 unique sites.

We then examined how many of the genes up- and downregulated in a *tup1* mutant harboured a Tup1p site of occupancy in their corresponding promoter region in wt. Out of the 555 genes differentially transcribed in the *tup1* mutant, 23% of these genes (131) contained a Tup1p peak (Fig 7B). Similarly, when we looked at the 933 genes differentially transcribed in the *cyc8* deletion mutant, 13% of these genes (125) contained a site of Cyc8p occupancy in their promoters (Fig 7C). Comparison of the unique sites of Tup1p and Cyc8p occupancy with the genes identified as being subject to unique repression by *TUP1* and *CYC8* respectively, revealed only a small overlap (S9 Fig). Thus, Tup1p and Cyc8p can be found at unique sites across the genome, and some of these sites correspond to those genes we previously described as being subject to unique *TUP1* and *CYC8* repression. However, overall, the correlation of Tup1p and Cyc8p occupancy with the genes up and down regulated in their absence, was poor.

### Summary of results

**Fig 8:**
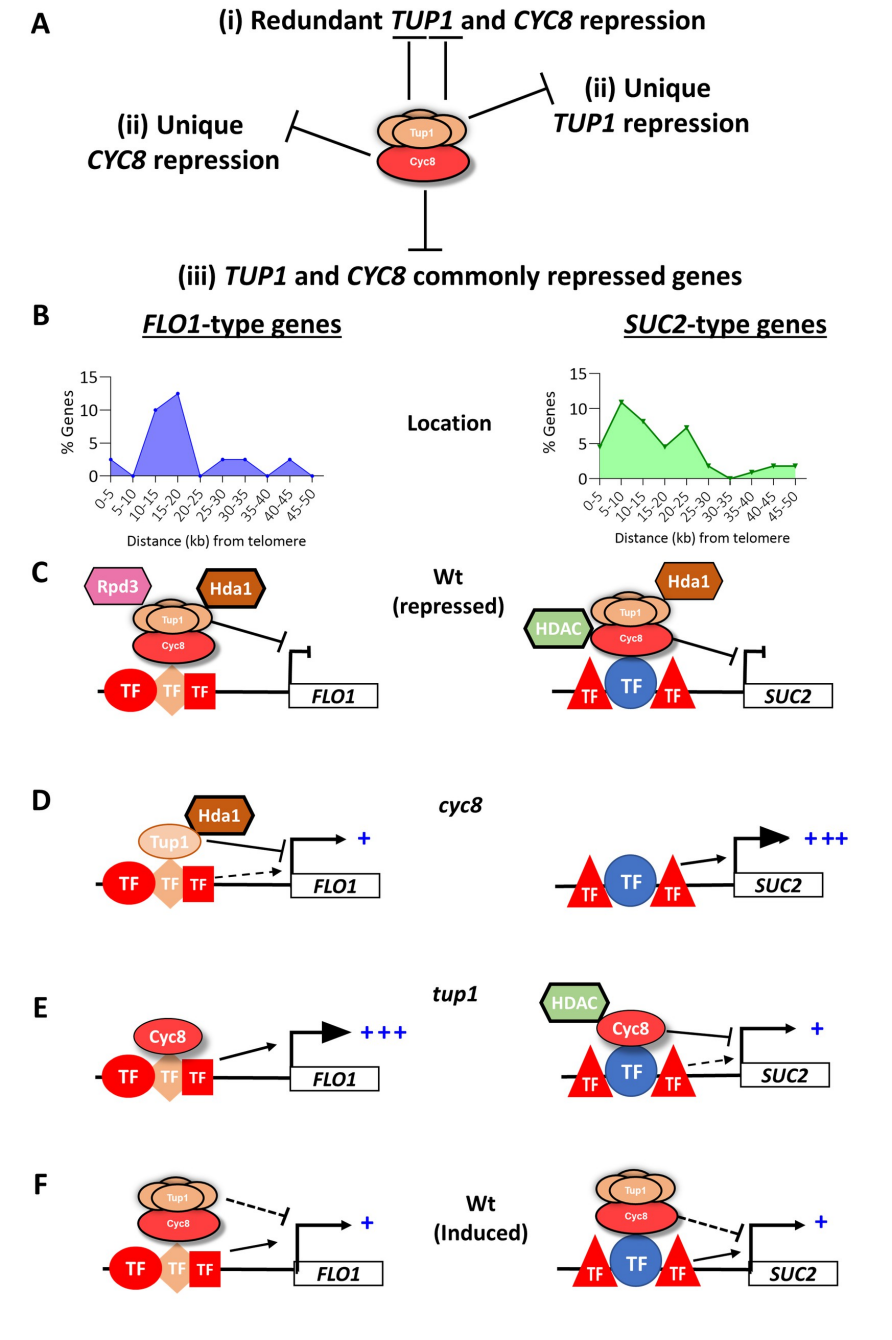
Summary of results and proposed ‘dimmer switch’ model for Tup1p and Cyc8p in the regulation of *FLO1*- and *SUC2*-type gene transcription. (A) RNA seq analysis revealed (i) genes subject to redundant repression by *TUP1* and *CYC8*, (ii) genes uniquely repressed by *CYC8* and *TUP1*, and (iii) *TUP1* and *CYC8* can make distinct contributions to commonly repressed genes. (B) The commonly repressed genes include genes which behave like *FLO1* (*FLO1*-type) and *SUC2* (*SUC2*-type) which are distributed differently within sub-telomeric regions. (C) *TUP1* and *CYC8* make the dominant contribution to *FLO1-* and *SUC2*-type gene repression respectively, in association with distinct HDACs (Rpd3p and Hda1p at *FLO1* via Tup1p; unknown HDACs at *SUC2* via putative interaction with Cyc8p) and transcription factors (TFs). (D) Tup1p occupies *FLO1* in the absence of Cyc8p to negatively influence *FLO1* in an Hda1p-dependent manner. (E) Cyc8p occupies *SUC2* in the absence of Tup1p to negatively influence *SUC2* via potential interaction with an uncharacterised HDAC. Occupancy of Cyc8p at *FLO1* in the *tup1* mutant has no role in repression. (F) Tup1-Cyc8 persists at both *SUC2* and *FLO1* when active to negatively modulate transcription, thus acting as a dimmer switch. (C-F) Strong and weak repressive roles of Tup1p/Cyc8p in transcription are depicted as solid and dashed flat-ended lines respectively. Putative strong and weak positive roles of TFs in transcription are shown as solid and dashed arrows respectively. Levels of low and high gene transcription are indicated by + and +++, respectively.

In summary, our analysis has revealed that (i) *CYC8* represses more genes than *TUP1*, (ii) some genes are uniquely repressed by either *TUP1* or *CYC8*, (iii) other genes are subject to redundant repression by *TUP1* and *CYC8*, and (iv), some *TUP1* repressed genes require *CYC8* for their de-repression and vice-versa (Fig 8A). We also show that *TUP1* and *CYC8* can make different contributions to commonly repressed genes (Fig 8B and 8C).

Many commonly repressed genes behave like *SUC2*, whereby *CYC8* makes the dominant contribution to repression (Fig 8C, *SUC2*-type genes). A smaller cohort of commonly repressed genes behave like *FLO1* and are subject to dominant repression by *TUP1* (Fig 8C, *FLO1*-type genes). These two cohorts of commonly repressed genes differ in their distribution within the HAST domains of sub-telomeric regions, in which they are enriched, and are associated with different transcription factor binding sites (Fig 8B and 8C). At *SUC2*-type genes, *CYC8* can exert repression in the absence of Tup1p, whereas at *FLO1*-type genes, *TUP1* can exert repression in absence of Cyc8p. In addition, *TUP1* and *CYC8* can exert repression at both *SUC2* and *FLO1* during gene activation.

Global ChIP data confirmed that Tup1p and Cyc8p can be found independent of each other, and at genes we had identified as being subject to unique *TUP1* and *CYC8*-dependent repression. In addition, Cyc8p could be detected at *SUC2* in the absence of Tup1p where it could directly negatively influence *SUC2* de-repression (Fig 8E). Furthermore, Tup1p could be detected at *FLO1* in the absence of Cyc8p, where it could directly negatively influence *FLO1* de-repression (Fig 8D). Our data also suggests that Tup1-Cyc8 persists at both the *SUC2* and *FLO1* genes when active, where it continues to negatively modulate transcription (Fig 8F).

Together, this suggests the potential for distinct novel regulatory roles for Tup1p and Cyc8p when functioning within, and possibly out with, the Tup1-Cyc8 complex. Furthermore, these data suggest the Tup1-Cyc8 complex can function as a molecular ‘dimmer switch’ to fine-tune active transcription in addition to its role as an outright repressor of transcription.

## Discussion

The Tup1-Cyc8 co-repressor complex was one of the first global repressors of gene transcription identified [1]. Several mechanisms of action have been proposed for Tup1-Cyc8 repression including the formation of repressive chromatin structures, inhibiting RNA polymerase II, and blocking transcription factor activation domains [37]. These roles have been proposed to function via the WD40 domain of Tup1p and the TPR motifs of Cyc8p which offer a versatile interface for multiple interactions with a large array of transcription factors, non-acetylated histone H3 and H4 tails, various histone deacetylases, and several RNA polymerase II subunits [8,11,58].

Most work to characterise Tup1-Cyc8 function used either *TUP1* or *CYC8* gene deletion mutants. This approach was justified considering the general model for Tup1-Cyc8 structure and function which predicts that a *tup1* mutant should lack the repressive activity of the complex, while the complex should be unable to bind target genes in a *cyc8* mutant [37]. Thus, both mutants should equally inhibit Tup1-Cyc8.

Our systematic analysis of single and double mutants deleted for *TUP1* and *CYC8* revealed the different strains have numerous distinct phenotypes and significant differences in their transcriptomes which has offered new insight into Tup1p, Cyc8p and Tup1-Cyc8 function. We propose that there are different subsets of Tup1-Cyc8 repressed genes which are subject to distinct regulation by either Tup1p or Cyc8p. Our data also suggests that Tup1p and Cyc8p can function independently within, and possibly out with, the complex.

Phenotypically, strains deleted for *CYC8* had the slowest growth and displayed a large cell morphology (Fig 1). Most strikingly, the *tup1* mutant had the strongest flocculation phenotype [46]. However, the flocculation phenotypes of the mutants did not always correlate with the cell’s responses to stress.

These data suggested that the wide-ranging differences in the *tup1* and *cyc8* mutants could be the result of altered transcription in strains deleted for *TUP1* and *CYC8*. In support of this, *TUP1* and *CYC8* made distinct contributions to the repression of *FLO1* and *SUC2* transcription, which are two genes known to be repressed by the Tup1-Cyc8 complex (Fig 2) [27,29,47]. *FLO1* transcription was de-repressed the most in the *tup1* mutant, whilst *SUC2* transcription was de-repressed the most in the *cyc8* mutant. This suggests a greater role for Tup1p in *FLO1* repression and a greater role for Cyc8p in *SUC2* repression. Furthermore, since *SUC2* de-repression in *tup1 cyc8* was greater than that in the *tup1* mutant, this suggests that *CYC8* exerts a repressive effect independent of Tup1p.

To determine whether the Tup1p independent role of *CYC8* at *SUC2* was direct or not, we examined Cyc8p occupancy at *SUC2* (Fig 6C). Surprisingly, we could not detect Cyc8p or Tup1p at *SUC2* in the glucose grown wt strain, where Tup1-Cyc8 has previously been detected (Fig 6B and 6C) [52,53]. However, we propose Tup1p and Cyc8p are present at the repressed *SUC2* promoter, but our ChIP protocol cannot detect them, possibly due to epitope masking by other factors present at this site. Conversely, a strong signal for Cyc8p could be detected at the *SUC2* promoter in the *tup1* mutant (Fig 6C). Thus, the transcription and ChIP results at *SUC2* in glucose grown cells are consistent with *CYC8* making the major contribution to *SUC2* repression and suggest that Cyc8p directly contributes to *SUC2* repression in the absence of Tup1p. This latter result suggests that at *SUC2*, under conditions of high glucose in the absence Tup1p, either Cyc8p can exert a repressive effect on its own, or the presence of Cyc8p at the *SUC2* promoter can enable another factor or factors to exert repression or inhibit activation. In support of this, recent work has shown that multiple proteins can interact with the Tup1-Cyc8 complex to fine-tune target gene transcription [6,7,59].

When we looked at *FLO1* regulation, *TUP1* played the greatest role in repression (Fig 2A). Furthermore, *TUP1* exerted a repressive effect in the absence of Cyc8p since *FLO1* de-repression was greater in *tup1 cyc8* compared to that in *cyc8*. Consistent with Tup1-Cyc8 dependent repression of *FLO1*, Tup1p and Cyc8p were detected at the wt *FLO1* promoter, when the gene is inactive (Fig 6E and 6F). In the *tup1* mutant, where *FLO1* transcription was de-repressed to the greatest extent, although Cyc8p occupancy was detected, our data suggests it does not contribute to repression. Surprisingly, we could detect Tup1p at *FLO1* in the *cyc8* mutant (Fig 6E). This suggests that Tup1p can directly negatively influence *FLO1* transcription in the absence of Cyc8p. This has not been reported before and could have been missed due to different normalisation strategies employed during ChIP analysis [27].

Analysis of *SUC2* transcription in the various mutants under conditions of low glucose revealed that *TUP1* and *CYC8* continue to exert a repressive effect upon *SUC2* transcription with *CYC8* again exerting the dominant effect (Fig 2B). Our data confirming that Tup1p and Cyc8p remain associated with the *SUC2* promoter under activation conditions of low glucose suggests that the complex is present at the active *SUC2* gene and acts as a ‘brake’ to dampen down on-going *SUC2* transcription (Fig 8F).

To examine *FLO1* gene activity under more physiological conditions, we reinstated the activator of *FLO1* transcription, Flo8p [55,56]. In a strain expressing Flo8p (the *FLO8+* strain), Flo8p occupied the *FLO1* promoter, and *FLO1* was transcribed at levels similar to that in a *cyc8* mutant (Fig 6G and 6H). Furthermore, Tup1p was also present at the active *FLO1* promoter in the *FLO8+* strain (Fig 6I). Importantly, since *FLO1* transcription in the *FLO8+* strain was similar to that in a *cyc8* mutant, this suggests that the presence of Tup1p, presumably in the context of the Tup1-Cyc8 complex, negatively influences on-going *FLO1* transcription.

Together, the *FLO1* and *SUC2* transcription and ChIP data suggest a model of action in which Tup1-Cyc8 represses the genes under non-inducing conditions (Fig 8C) and persists at both genes during their activation to negatively modulate on-going transcription (Fig 8F). The Tup1-Cyc8 occupancy at the actively transcribed genes could also poise genes for rapid repression.

The persistence of Tup1-Cyc8 at active genes has been reported previously and provided evidence of Tup1-Cyc8 acting as an activator of gene transcription [20,21,35,52,53]. At the repressed *GAL1* gene, Tup1-Cyc8 occupancy enabled recruitment of the SAGA coactivator complex which was proposed to disrupt Tup1-Cyc8 repression and aid *GAL1* transcription under inducing conditions [52]. Tup1-Cyc8 was also shown to prime the repressed mating-type specific genes under its negative control for activation via Gcn5p-dependent pre-acetylation of histones at the repressed gene promoters [60]. Interestingly, a recent study revealed that activator-dependent eviction of *HO* promoter nucleosomes was required for Tup1p to bind to the promoter and bring about repression [61]. Hence, Tup1-Cyc8 is emerging as being central to the dynamic interplay between gene repression and activation in which repressors are required for activation and activators are required for repression.

Our RNA-seq analysis confirmed that most genes under control of *TUP1* and *CYC8* were negatively regulated, consistent with Tup1-Cyc8 primarily being considered to be a repressor of gene transcription (Fig 3A) [2,3]. We also confirmed that the complex can positively regulate gene transcription, although far fewer genes require *TUP1* and *CYC8* for activation.

We therefore limited our analysis of the RNA seq data to the genes upregulated in the *tup1*, *cyc8* and *tup1 cyc8* mutants where it could be inferred that *TUP1* and *CYC8* played a role in repression. The data showed that almost twice as many genes were upregulated in the *cyc8* mutant (809) compared to the *tup1* mutant (469), suggesting a wider role for Cyc8p in global gene repression. Of the genes upregulated in the *tup1*, *cyc8*, and *tup1 cyc8* mutants, there was a cohort of 429 genes that were commonly de-repressed in all the mutants (Fig 3B). We propose that these genes are subject to the most robust repression by Tup1p and Cyc8p functioning as the Tup1-Cyc8 complex.

Analysis of the *TUP1* and *CYC8* commonly repressed genes revealed two distinctly regulated classes of genes (Fig 4C). A large number of genes behave like *SUC2* and were most repressed via *CYC8*, whilst a smaller subset behaves like *FLO1* and were most repressed by *TUP1* (Fig 8C).

We showed that the 429 commonly repressed genes were enriched in gene-sparse sub-telomeric regions in an area that correlated well with the zones subject to the most Hda1p activity; the so-called HAST domains (Fig 5A and 5C) [27,62]. Interestingly, the *FLO1*-type genes were enriched within a single narrow peak within the HAST domain, whilst the *SUC2*-type genes showed a broader distribution either side of the peak of *FLO1*-type genes (Figs 5D and S4). This observation reinforces the link between Tup1-Cyc8 repression activity and histone deacetylases (HDACs) and might suggest a different HDAC dependency of the *SUC2*-type and *FLO1*-type genes [25,27].

In support of this hypothesis, it has been shown that Tup1p and Cyc8p have distinct interaction profiles with various class I and II HDACs [26,63]. For example, Rpd3p can interact with Cyc8p independently of Tup1p, whereas Hda1p has been shown to physically interact with Tup1p. At *FLO1*, repression requires both Hda1p and Rpd3p and both HDACs occupy the inactive *FLO1* promoter in a Tup1-Cyc8 dependent manner [27]. Interestingly, Hda1p, but not Rpd3p, remains detectable at the partially de-repressed *FLO1* promoter in a *cyc8* mutant [27]. Furthermore, when *hda1* is additionally deleted in a *cyc8* mutant, the level of *FLO1* de-repression is elevated to a level similar to that in a *tup1 cyc8* mutant (S12C Fig; compare *cyc8*, *hda1 cyc8* and *tup1 cyc8*). Thus, the Tup1p that we have detected at the *FLO1* promoter in the *cyc8* mutant could mediate the reported retention of Hda1p which could contribute to the Cyc8p independent repression of *FLO1* transcription shown here (Fig 8D).

It might therefore be predicted that a different HDAC would be detected at the *SUC2* promoter in a *tup1* mutant having been retained via the observed occupancy of Cyc8p at the *SUC2* promoter in the absence of Tup1p (Fig 8E). Thus, it might be that the *FLO1*-type genes are most dependent upon Hda1p for repression via interaction with Tup1p, and the *SUC2-*type genes might require an alternative HDAC for most repression via interaction with Cyc8p (Fig 8C). To confirm this, HDAC occupancy in wt, *tup1* and *cyc8* mutant backgrounds would need to be performed, and global Tup1p and Cyc8p occupancy profiles in the respective *cyc8* and *tup1* mutants identified and analysed.

Previous work had shown that the *FLO1* and *SUC2* genes were subject to long-range chromatin remodelling in their extensive upstream intergenic regions [28–30]. Although we could not identify any significant difference in the length of intergenic region upstream of the *SUC2*- and *FLO1*-type genes, there was a positive correlation between the level of gene de-repression and the length of the upstream regions in those mutants yielding maximal de-repression (S6C and S6D Fig). Thus, although there is an influence of the length of upstream region upon *TUP1* and *CYC8*-dependent gene repression, this effect is common to both the *FLO1*- and *SUC2*-type genes. This supports a role for long-range chromatin remodelling in mediating Tup1-Cyc8 target gene repression [28,29].

Analysis of transcription factor binding motifs upstream of the *SUC2*- and *FLO1*-type genes, revealed that the *FLO1*-type genes had a larger proportion of Yap1p, Hac1p and Gcn4p binding motifs whilst Hap1p, Nrg1p and Msn2p/Msn4p were most enriched at the *SUC2*-type genes (S1 Table). Both Hac1p and Yap1p can recruit Tup1-Cyc8 to target genes where they play a positive role in transcription, and Hac1p has been shown to physically interact with Cyc8p [64,65]. This correlates with the higher transcription of the *FLO1* type genes in the *tup1* mutant strain compared to that in the *cyc8* mutant strain if the Cyc8p occupancy detected at *FLO1* in the *tup1* mutant is also found at all the *FLO1*-type genes (Fig 8E). Gcn4p also plays a role in the activation of transcription. At *ARG1*, Cyc8p was needed for efficient binding of Gcn4p to the promoter, whereas loss of *TUP1* had less of an effect on Gcn4p binding [59]. Again, this correlates with the lower de-repression of the *FLO1*-type genes in a *cyc8* mutant compared to de-repression in a *tup1* mutant and the Cyc8p occupancy detected at *FLO1* in the absence of Tup1p (Fig 8D and 8E).

Together, our analysis suggests that the two cohorts of commonly repressed genes have differences in transcription factor (TF) binding and HDAC dependency. The HDACs might contribute most to gene repression, whilst the TFs might be more relevant to target genes when active. Importantly, we suggest that Tup1-Cyc8 persists at active target genes with the Tup1p and Cyc8p subunits interacting and influencing HDAC and TF occupancy and activity differently at the distinct gene cohorts to yield the transcriptional outcome.

Our analysis also found subsets of genes uniquely upregulated in either the *tup1* or *cyc8* mutants (Fig 3B). This suggests Tup1p and Cyc8p could have independent repressive roles at genes when residing within the complex or when functioning independently out with the complex. Indeed, global occupancy analysis reveals Tup1p and Cyc8p can be found at 340 and 85 unique sites across the genome, respectively (Fig 7A). Furthermore, the Tup1p independent occupancy of Cyc8p at *SUC2*, where it can exert a repressive role, supports the fact that Cyc8p can influence repression independent of Tup1p (Fig 6C). Similarly, the Cyc8p independent occupancy of Tup1p at *FLO1* supports a direct role for Tup1p contributing to gene repression in the absence of Cyc8p (Fig 6E).

The correlation of Tup1p and Cyc8p occupancy with the genes under their control was poor (Fig 7B and 7C). This suggests either most genes are indirectly regulated by Tup1p and Cyc8p or, that current ChIP analysis of Tup1p and Cyc8p cannot identify all their binding sites. Application of improved techniques to measure protein occupancy may reveal their precise locations across the genome in the future. Additionally, analysing cells under dynamic growth conditions might allow the entire suite of sites occupied by Tup1p and Cyc8p, and the genes under their control, to be fully exposed [66].

By comparing transcription profiles in the single mutants to the double mutant we identified genes subject to redundant repression, and some genes at which de-repression in *cyc8* was *TUP1*-dependent, and vice versa. (Fig 3B). Both results suggest novel functions for Tup1p and Cyc8p which are inconsistent with the model for Tup1-Cyc8 complex function. Despite the caveats described above, ChIP analysis of Tup1p occupancy in a *cyc8* mutant and Cyc8p occupancy in a *tup1* mutant might confirm what our global transcription analysis has suggested.

In summary, our analysis offers a compendium of data for *tup1* and *cyc8* mutants to be considered when studying Tup1-Cyc8 complex activity by traditional gene deletion analysis. We have shown *TUP1* and *CYC8* can make distinct contributions to repression and activation of specific gene cohorts. We show evidence that *TUP1* and *CYC8* can repress genes independently of each other and offer evidence that, at least at *FLO1* and *SUC2* respectively, this repression can be direct. Our data suggests that the mechanism of action of Tup1-Cyc8 is much more complex than previously thought and that Tup1p and Cyc8p can have individual roles which may be functioning when the proteins are residing within and possibly out with the complex. Further study will be required to fully elucidate the roles of the Tup1p and Cyc8p proteins and the Tup1-Cyc8 complex. Indeed, the cumulative evidence shown here and elsewhere points to the Tup1-Cyc8 complex having a much more versatile role in gene regulation which is not limited to it functioning solely as a repressor [23].

## Methods

### Yeast strains and growth conditions

The *S*. *cerevisiae* strains used are described in S2 Table. Yeast gene deletions and tagging were performed using polymerase chain reaction (PCR)-based methods [67,68]. All strain constructions were confirmed by PCR and/or western blot analysis and assayed for appropriate phenotypes/function where relevant. The construction and confirmation of the restored *FLO8* gene and the *FLO8-*myc strain, are described in S7 and S8 Figs. Cells were grown at 30°C in YPD medium unless stated otherwise.

### Protein analysis

Protein lysate preparation and western blotting analysis were performed as previously described [27,54]. The antibodies and conditions used are described in S3 Table.

### Flocculation assay

This assay was performed as previously described [54,69]. Cells with a flocculant phenotype aggregate in the absence of EDTA and are dispersed in the presence of EDTA (final concentration of 2 mM).

### Cell microscopy

Exponentially growing cells were resuspended in either sterile water or EDTA (2 mM) and viewed under 100x oil immersion magnification. Leica Application Suite (LAS) software was used to capture the images.

### Survival assays

The survival assays were performed as previously described with minor adaptations [46,70]. Aliquots (1 ml) of 5 x 10^8^ exponentially growing cells were resuspended in 5 ml YPD and incubated for 1 hour at 30°C. Aliquots were exposed to ethanol (15%), hydrogen peroxide (5 mM), Amphotericin B (15 μg/ml), growth at 50°C, and growth at 30°C (control) for 1 hour. The percent survival compared to the control was calculated by counting colony forming units (CFUs).

### Determination of transcription factor binding motifs

The known DNA binding motifs for all transcription factors (TF) found in the sequences 1000 bp upstream of the *FLO1*- and *SUC2*-type genes were retrieved from YEASTRACT [71]. The number of times a motif was found upstream of each gene was calculated and this information was compiled into a table (S1 Table). For the final analysis, any TF DNA binding motif that was found at less than 40% of both the *FLO1* and *SUC2*-type genes was omitted. A parameter of at least a difference of 10% in the proportion of genes with that motif in each of the groups (*FLO1*-type and *SUC2*-type genes) was categorised as a significant difference.

### RT-qPCR

RNA extraction, cDNA preparation and RT-qPCR analysis were performed as previously described [54]. For *SUC2* analysis, exponentially growing YPD cultures were divided into two equal portions. Cell pellets were washed twice and resuspended in YP containing glucose at either 2% (repressed) or 0.05% (de-repressed). Cultures were incubated for a further 120 min [29]. Values were normalised to *ACT1* RNA. Primers used are shown in S4 Table.

### RNA-Seq

RNA was extracted from exponentially growing cells using the Hot Phenol method and purified using the RNeasy Minelute Cleanup Kit (Qiagen) [72]. Total RNA was sent to Genewiz (Azenta Life Sciences) for rRNA depletion, cDNA library preparation and strand-specific RNA sequencing using the Illumina HiSeq Platform. Sequence reads were trimmed to remove possible adapter sequences and nucleotides with poor quality using Trimmomatic v.0.36. The trimmed reads were mapped to the *Saccharomyces cerevisiae* S288c reference genome available on ENSEMBL using the STAR aligner v.2.5.2b. and BAM files were generated. Unique gene hit counts were calculated by using featureCounts from the Subread package v.1.5.2. Genewiz preformed DGE analysis using the DESeq2 Bioconductor package. Using normalised counts, Log_2_ fold change and a Benjamini-Hochberg adjusted p-value was then calculated. Conversion from BAM into BigWig files was carried out using bamCoverage from the deeptools package [73]. The Bigwig files, which represents coverage of mapped reads, were uploaded to JBrowse and are available to view at http://bioinf.gen.tcd.ie/jbrowse/?data=RNA-seq_Tup1Cyc8_merged. The RNA-seq datasets are available in the Gene Expression Omnibus (GEO) repository (accession code GSE230732). S1 Appendix provides the RNA-seq data used to construct Figs 3, 4, 5, 7, S4, S5, S6 and S9.

### Chromatin immunoprecipitation (ChIP)

Locus-specific ChIP was performed as previously described [27,54]. The antibodies and conditions used are shown in S5 Table. For ChIP, occupancy signals were determined by comparing the enrichment of DNA found in the immunoprecipitated (IP) material versus the input (IN) material. This (IP/IN) signal was then normalised to an IP/IN signal at an internal negative control region (*Tel-VI* for RNAP II, *Int-V* for Tup1p, Cyc8-Myc and Flo8-Myc) to give the ‘relative occupancy’. Tup1p and Cyc8-Myc relative occupancy were further normalised to similarly processed ChIP results from a *tup1* deletion and an untagged (No tag) strain, respectively. Details to show the ChIP resolution and the normalisation pathway are shown in S10 and S11 Figs. Primers used are shown in S4 Table.

The global Tup1p and Cyc8p occupancy data used in Fig 7, and the Rsc1p and Gcn5p data used in S4B Fig was retrieved from a ChIP-Exo analysis performed by Rossi *et al*., 2021 [57], and is available in S2 Appendix. The processed ChIP-Exo peaks for Tup1p and Cyc8p occupancy were uploaded to JBrowse (peak mid-points are shown) and can be viewed at http://bioinf.gen.tcd.ie/jbrowse/?data=RNA-seq_Tup1Cyc8_merged.

## Supporting information

S1 Appendix Lists of genes up and downregulated in the *cyc8*, *tup1* and *tup1 cyc8* mutants and values of fold-changes in transcription relative to wt. The Excel file contains the RNA-seq data used to construct the appropriate figures.
(XLSX)

S2 Appendix Lists of sites of Tup1p, Cyc8p, Rsc1p and Gcn5p occupancy. Data retrieved from Rossi *et al*., 2021 [57] and used in Figs 7 and S4B.
(XLSX)

S3 Appendix Values for data used to create graphs in the figures. The Excel file contains multiple tabs, with each tab containing the data for a single figure.
(XLSX)

S1 Fig *FLO5*, *FLO9* and *FLO10* transcript levels measured by RT-qPCR. mRNA values in the strains indicated were normalised to *ACT1* mRNA and error bars reflect standard deviation from 3–4 biological replicates.
(TIF)

S2 Fig RT-qPCR validation of RNA-Seq data. (A) JBrowse image of RNA-Seq data of *SUR2* mRNA levels in wt, *tup1*, *cyc8* and *tup1 cyc8* strains. (B) RT-qPCR analysis of *SUR2* mRNA levels in wt and each of the mutant strains. (C) JBrowse image of RNA-Seq data of *PHO3* mRNA levels in wt, *tup1*, *cyc8* and *tup1 cyc8* strains. (D) RT-qPCR analysis of *PHO3* mRNA levels. In both B and D, mRNA levels were normalised to *ACT1* mRNA and error bars reflect standard deviation (* represents a p-value of p<0.05, ** represents a p-value of p<0.005 determined by a One-way ANOVA analysis, n = 3). (E) JBrowse image of RNA-Seq data of *FIT2* mRNA levels in wt, *tup1*, *cyc8* and *tup1 cyc8* strains. (F) JBrowse image of RNA-Seq data of *MET6* mRNA levels in wt, *tup1*, *cyc8* and *tup1 cyc8* strains.
(TIF)

S3 Fig Regulation of *RNR3* transcription. (A) RNA polymerase II (RNAP II) occupancy at the *RNR3* open reading frame (ORF) in wt, *tup1*, *cyc8* and *tup1 cyc8* in glucose grown cells measured by chromatin immunoprecipitation (ChIP). RNAP II signals (IP/IN) were normalised to an internal negative control region (IP/IN at *Tel-VI*) (n = 3). (B) *RNR3* transcript levels measured relative to *ACT1* mRNA levels using RT-qPCR in the strains indicated (n = 3). In A and B, error bars reflect standard deviation. (C) JBrowse image of RNA-Seq data of *RNR3* mRNA levels in wt, *tup1*, *cyc8* and *tup1 cyc8* strains.
(TIF)

S4 Fig Distribution of *FLO1*- and *SUC2*-type genes across the genome. (A) Distribution of the 429 commonly upregulated genes over the first 100 kb regions from the telomeres divided into 5 kb regions and separated into *FLO1*-type (blue), *SUC2*-type (green), and the remaining commonly upregulated genes (pink). The percentage of genes in each group was calculated. (B) Distribution of Gcn5p and Rsc1p occupancy over the first 100 kb regions from the telomeres divided into 5 kb regions. Occupancy of each protein in each 5 kb region is shown as a % of total protein occupancy over this region. It is important to note that Gcn5p and Rsc1p do not co-localise with the sub-telomeric sites of enrichment of the *FLO1*- and *SUC2*-type genes, thus acting as negative controls to support the observation of the exclusive *FLO1*- and *SUC2*-type gene localization as being unique to Tup1p and Cyc8p regulated genes. Gcn5p and Rsc1p occupancy data were extracted from Rossi *et al*., 2021 [57].
(TIF)

S5 Fig *FLO1-* and *SUC2*-type genes are equally robustly repressed in wt. Scatterplot to show the average transcript per million (TPM) values from the three biological wt replicates for the *FLO1*- and *SUC2*-type genes. We assigned a cut-off of average TPM values ≤10 as ‘off’ in wt, represented by the dashed line.
(TIF)

S6 Fig Analysis of *FLO1*- and *SUC2*-type gene intergenic region length and ORF size. (A) Schematic depicting how intergenic length was calculated. (B) Scatterplot depicting the up- and downstream intergenic lengths of *FLO1-* and *SUC2*-type genes and the closest protein coding gene; also shown are the ORF lengths of *FLO1-* and *SUC2-*type genes (from SGD) (75). **Correlating *FLO1*- and *SUC2*-type gene upstream intergenic region length with gene de-repression.** Graphs depicting the length of the upstream intergenic length (X axis) and the change in transcription compared to wt (Y axis) for the (C) *FLO1-*type and (D) *SUC2-*type genes in *tup1*, *cyc8* and *tup1 cyc8* mutant strains. A line of best fit is shown for each strain. For *FLO1*-type genes (C), a two tailed Spearman correlation showed a statistically significant correlation between the upstream intergenic length and upregulation of transcription in the *tup1 cyc8* strain compared to wt (P = 0.0294). For *SUC2*-type genes (D), a two tailed Spearman correlation showed a significant correlation in the *cyc8* strain (p = 0.0449).
(TIF)

S7 Fig Restoration of a functional *FLO8* gene by PCR-mediated mutagenesis. (A) In the first round of mutagenesis, a PCR product containing a selectable marker integrates into the position immediately downstream of the *FLO8* 3’ ORF by homologous recombination. The resulting strain contains a genomic copy of *FLO8* immediately followed by the marker. (B) A forward primer was designed with homology to the *FLO8* 5’ ORF that contained an A-G base-pair substitution corresponding to position +425 of the *FLO8* ORF. This was used in conjunction with a reverse primer with homology to an intergenic region downstream of *FLO8*. Using genomic DNA from the strain with the selectable marker directly downstream of the *FLO8* ORF; these primers were used to generate a PCR product that contained the majority of the *FLO8* ORF, but with the A-G point mutation at position +425. This product also contained the selectable marker adjacent to the *FLO8* 3’ ORF. (C) A second transformation was carried out in a wild type BY4741 strain using the PCR product containing the point mutation at position +425 in the *FLO8* ORF. This resulted in a strain with a genomic copy of *FLO8* containing a G at position +425 in place of an A. This strain also contained a selectable marker immediately downstream of the *FLO8* ORF. The resulting strain (YMC19, *FLO8*+) was sequenced to confirm the point mutation. The restored *FLO8* gene in YMC19 was subsequently tagged with a 9-Myc epitope to generate strain YMC34.
(TIF)

S8 Fig Confirmation of *FLO8* expression, *FLO8* function, and Flo8-Myc occupancy in the *FLO8+* strain. (A) Transcription from the non-functional *FLO8* gene in wt and *cyc8*, and from the restored *FLO8* gene in YMC19 (*FLO8+*), was analysed by RT-qPCR (error bars represent SD, n = 2). (B) Expression of Flo8-Myc in YMC34 was confirmed by western blot analysis. Wt and a Cyc8-myc strain were used as controls. Actin was used as a loading control. The proteins detected for Cyc8-Myc and Flo8-Myc were of the expected size. (C) *FLO1* and (D) *SUC2* mRNA levels detected by RT-qPCR in wt, *cyc8* and the *FLO8*+ strain. The result shows that *FLO1* is transcribed in the *FLO8*+ strain, whilst *SUC2* is not transcribed (error bars represent SD, n = 2). The data in C is the same data shown in Fig 6G. (E) Flo8-Myc occupancy (IP/in) at the *FLO1* promoter, *SUC2* promoter, and at a negative control region, *Int-V*. (F) Flo8-Myc occupancy data from (E) shown as ‘relative occupancy’ following normalisation to *Int-V* (error bars represent SD, n = 2). The Flo8-Myc relative occupancy data shown here for *FLO1* is the same data shown in Fig 6H. The results in C-F show that the impact of Flo8-Myc expression are specific for *FLO1*; Flo8-Myc does not occupy the *SUC2* promoter, and *SUC2* remains repressed in the *FLO8*+ strain.
(TIF)

S9 Fig Overlap of Tup1p and Cyc8p occupancy with genes subject to unique *TUP1* and *CYC8* repression. (A) Venn diagram showing the overlap between global Tup1p occupancy and genes identified as being uniquely repressed by *TUP1* (see Fig 3F). (B) Venn diagram showing the overlap between global Cyc8p occupancy and genes identified as being uniquely repressed by *CYC8* (see Fig 3D). Tup1p and Cyc8p occupancy data were retrieved from Rossi *et al*., 2021 [57].
(TIF)

S10 Fig Resolution capacity of ChIP analysis. (A) Representative agarose gel to show DNA fragment size before and after chromatin sonication. A 1 kb DNA ladder and a 100 bp ladder (NEB) are indicated. The gel shows results from two wt samples (wt-1 and wt-2). For each sample the DNA included was 1: pre-sonicated input genomic DNA (RNase treated); 2: pre-sonicated input genomic DNA (RNase and DNase treated); 3: sonicated input DNA (RNase treated); 4: sonicated input DNA (RNase and DNase treated). (B) Schematic of the amplicons used for ChIP analysis across the *FLO1* promoter and at the *Tel-VI* negative control region. (C) Cyc8-Myc occupancy across the *FLO1* promoter region to show specific enrichment at -585 bp in wt and *tup1*. Cross linked chromatin from a Cyc8-Myc strain (wt), *tup1*/Cyc8-Myc strain (*tup1*), and an untagged strain (No tag) were immunoprecipitated with antibodies against the Myc tag. IP/IN for each of the indicated amplicons, as well as a negative control region within the right arm of *Tel-VI*, are shown. Mean and standard deviation are shown, * = p≤0.05, ** = p≤0.005 obtained from One-Way ANOVA analysis (n = 3–4).
(TIF)

S11 Fig Examples to show ChIP data normalisation. (A) Schematic to illustrate the amplicons used for ChIP analysis at the *FLO1* promoter and ORF. (B-C) RNA polymerase II (RNAP II) occupancy at the *FLO1* ORF in wt, *tup1*, *cyc8* and *tup1 cyc8* mutant strains. (B) IP/ IN for RNAP II occupancy at the *FLO1* ORF and the negative control region (*Tel-VI*) are shown. (C) RNAP II occupancy at the *FLO1* ORF normalised to *Tel-VI* to yield ‘relative occupancy’. (D-F) Cyc8-Myc occupancy at the *FLO1* promoter. Chromatin from a Cyc8-Myc strain (wt), a *tup1*/Cyc8-Myc strain (*tup1*), and an untagged strain (No tag), were immunoprecipitated with antibodies against the Myc tag. (D) IP/IN for Myc occupancy at the *FLO1* promoter and the negative control region (*Tel-VI)*, are shown. (E) Cyc8-Myc occupancy at the *FLO1* promoter normalized to occupancy at *Tel-VI* to yield ‘relative occupancy’. (F) *Cyc8*-Myc relative occupancy following normalization to relative occupancy in the No Tag strain. In all graphs mean and standard deviation are shown, * = p≤0.05, ** = p≤0.005 obtained from One-Way ANOVA analysis (n = 3–6).
(TIF)

S12 Fig Loss of *CYC8* and *HDA1* specifically abolishes repression of *FLO1* transcription. (A) PCR results from genomic DNA of the strains indicated to confirm the *hda1*::*KAN* mutant strain. Lane 1: 10 bp marker (NEB), lane 2, 3: PCR using primers for *ACT1* (positive PCR control), lanes 4, 5: PCR using primers upstream of *HDA1* (hda1DFconF) and internal to *hda1*::*KAN* (KanB), lanes 6, 7: PCR using primers downstream of *HDA1* (hda1DFconR) and internal to *hda1*::*KAN* (KanC). (B) Western Blot analysis to confirm the deletion of *CYC8* in a *hda1* mutant strain (in duplicate). β-actin was used as a loading control. Bands detected were of the expected size. RT-qPCR analysis of transcription of (C) *FLO1*, (D) *SUC2* and (E) *PMA1* in the strains indicated. Values were normalised to *ACT1* mRNA and error bars reflect standard deviation (n = 3–4). (RT-qPCR data of *FLO1* and *SUC2* in the wt, *tup1*, *cyc8* and *tup1 cyc8* strains has previously been shown in Fig 2). (F) Schematic to depict Tup1p-dependent role of Hda1p in *FLO1* repression. In a *cyc8* mutant strain we propose that Hda1p, in association with Tup1p, represses *FLO1* in the absence of *CYC8*. Loss of both *CYC8* and *HDA1* results in high *FLO1* de-repression (compare *FLO1* mRNA levels in *hda1 cyc8* and *tup1 cyc8*). This result is specific to *FLO1*. Transcription at *SUC2* and *PMA1* are not significantly affected.
(TIF)

S1 Table Transcription factor DNA binding motifs upstream of *FLO1*- and *SUC2*-type genes. (DOCX)

S2 Table Yeast strains used in this study. (DOCX)

S3 Table Antibodies used in Western immunoblotting. (DOCX)

S4 Table Oligonucleotides used in study. (DOCX)

S5 Table Antibodies and conditions used for chromatin immunoprecipitation (ChIP). (DOCX)
