# Supplementary figures and images for: Systematic analysis of tup1 and cyc8 mutants reveals distinct roles for TUP1 and CYC8 and offers new insight into the regulation of gene transcription by the yeast Tup1-Cyc8 complex
Source: PLoS Genet. 2023 Aug 11;19(8):e1010876. doi: 10.1371/journal.pgen.1010876 (PMC10446238; doi:10.1371/journal.pgen.1010876)

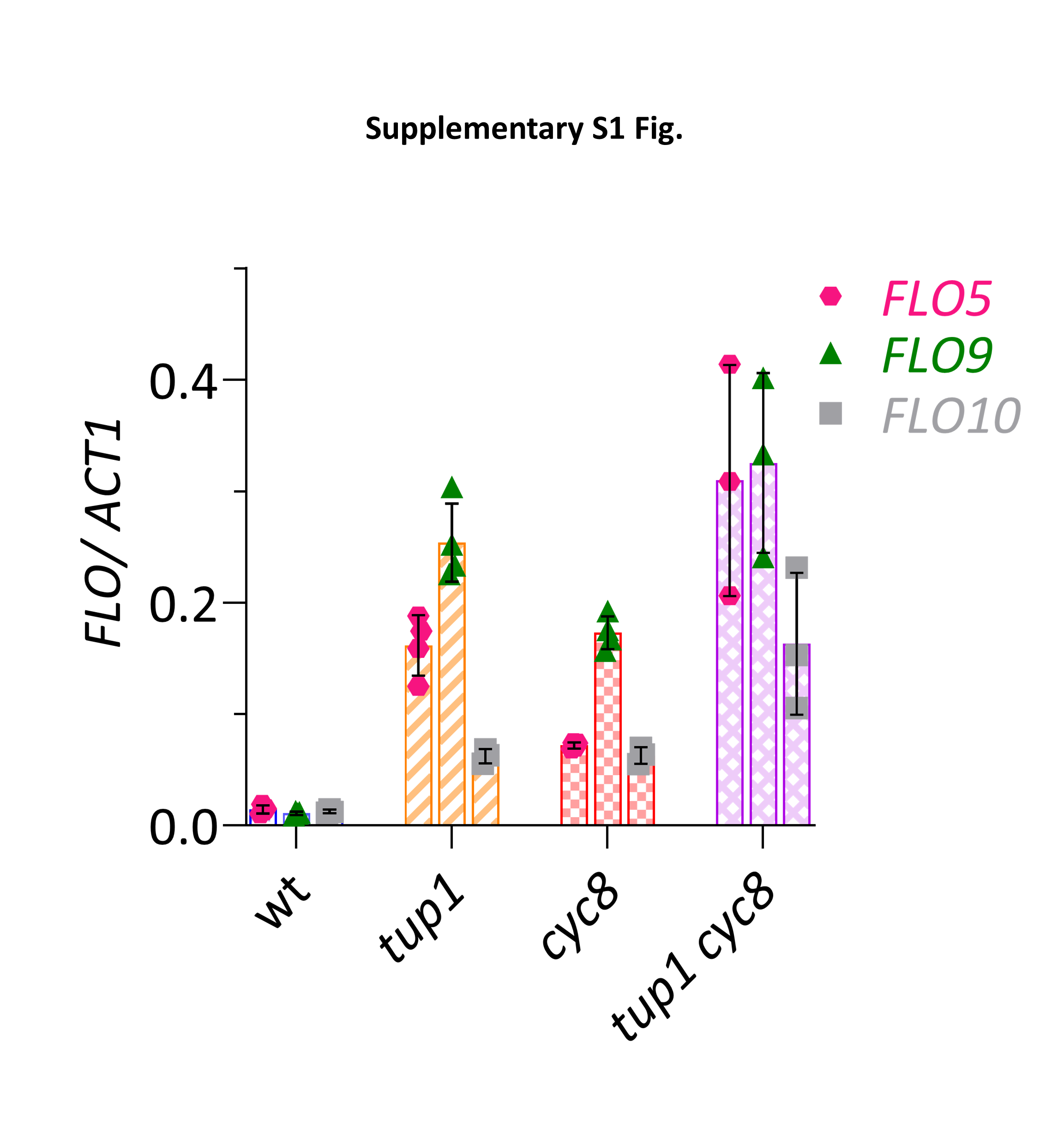

Supplement: S1 Fig — mRNA values in the strains indicated were normalised to ACT1 mRNA and error bars reflect standard deviation from 3–4 biological replicates. (TIF) [file pgen.1010876.s004.TIF]

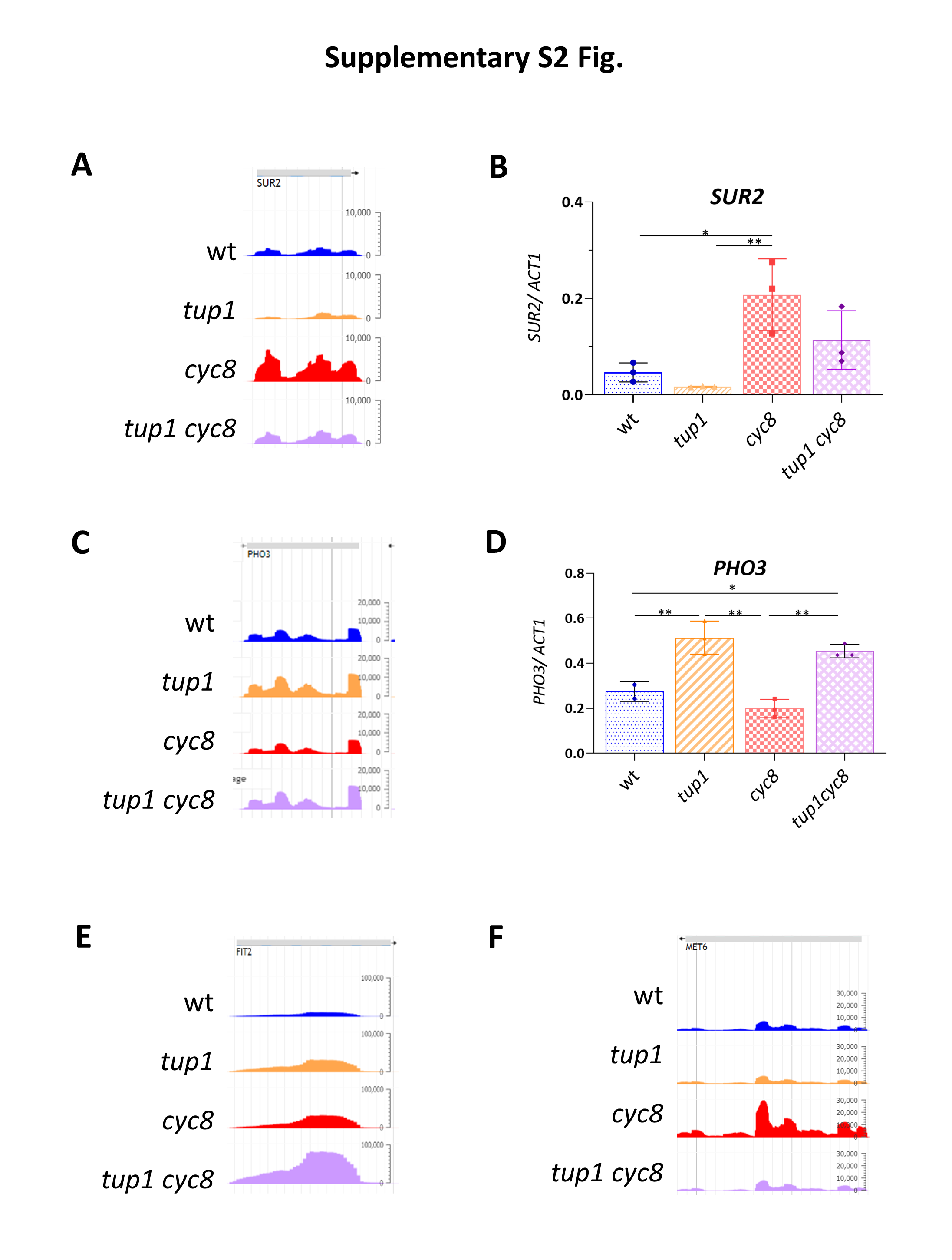

Supplement: S2 Fig — (A) JBrowse image of RNA-Seq data of SUR2 mRNA levels in wt, tup1, cyc8 and tup1 cyc8 strains. (B) RT-qPCR analysis of SUR2 mRNA levels in wt and each of the mutant strains. (C) JBrowse image of RNA-Seq data of PHO3 mRNA levels in wt, tup1, cyc8 and tup1 cyc8 strains. (D) RT-qPCR analysis of PHO3 mRNA levels. In both B and D, mRNA levels were normalised to ACT1 mRNA and error bars reflect standard deviation (* represents a p-value of p<0.05, ** represents a p-value of p<0.005 determined by a One-way ANOVA analysis, n = 3). (E) JBrowse image of RNA-Seq data of FIT2 mRNA levels in wt, tup1, cyc8 and tup1 cyc8 strains. (F) JBrowse image of RNA-Seq data of MET6 mRNA levels in wt, tup1, cyc8 and tup1 cyc8 strains. (TIF) [file pgen.1010876.s005.TIF]

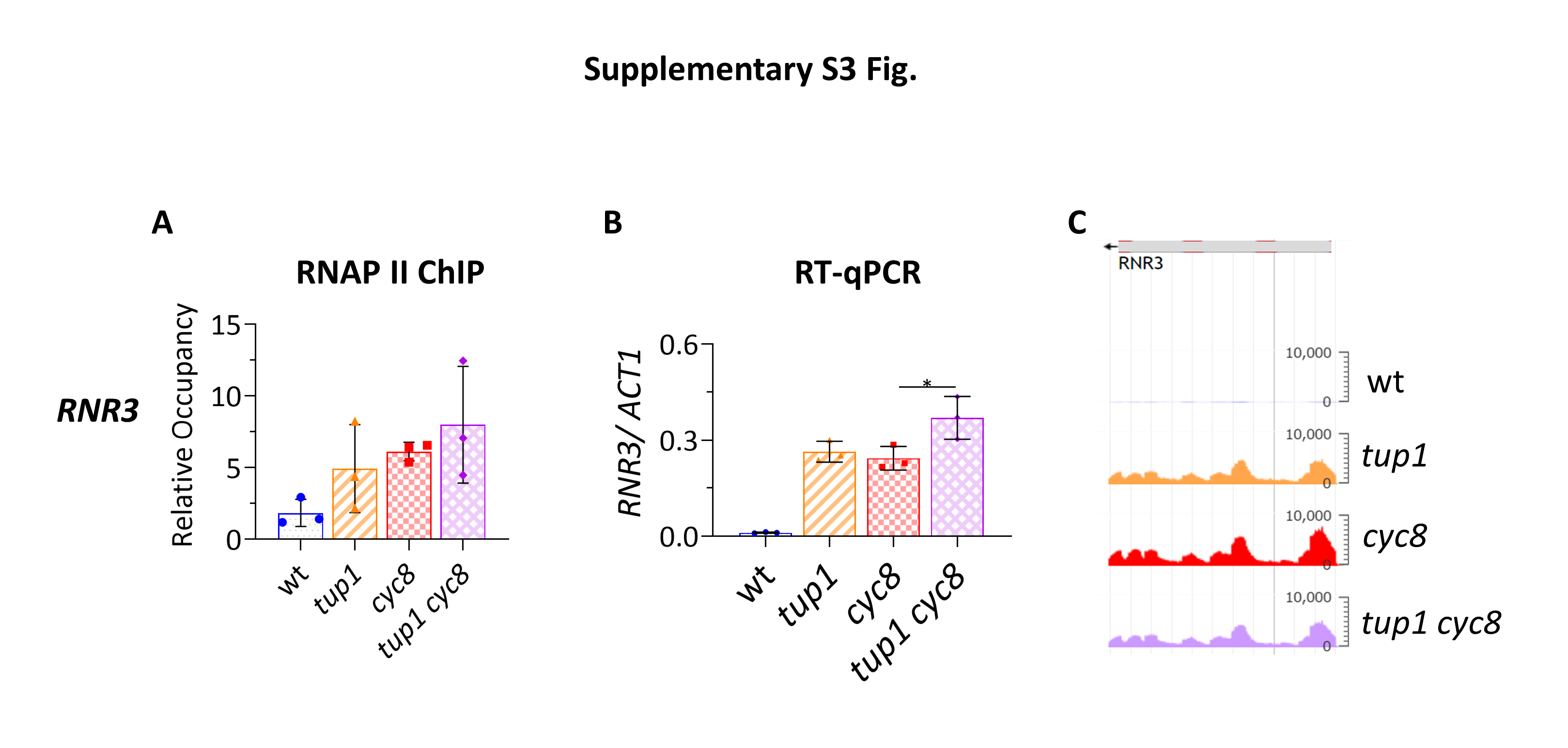

Supplement: S3 Fig — (A) RNA polymerase II (RNAP II) occupancy at the RNR3 open reading frame (ORF) in wt, tup1, cyc8 and tup1 cyc8 in glucose grown cells measured by chromatin immunoprecipitation (ChIP). RNAP II signals (IP/IN) were normalised to an internal negative control region (IP/IN at Tel-VI) (n = 3). (B) RNR3 transcript levels measured relative to ACT1 mRNA levels using RT-qPCR in the strains indicated (n = 3). In A and B, error bars reflect standard deviation. (C) JBrowse image of RNA-Seq data of RNR3 mRNA levels in wt, tup1, cyc8 and tup1 cyc8 strains. (TIF) [file pgen.1010876.s006.TIF]

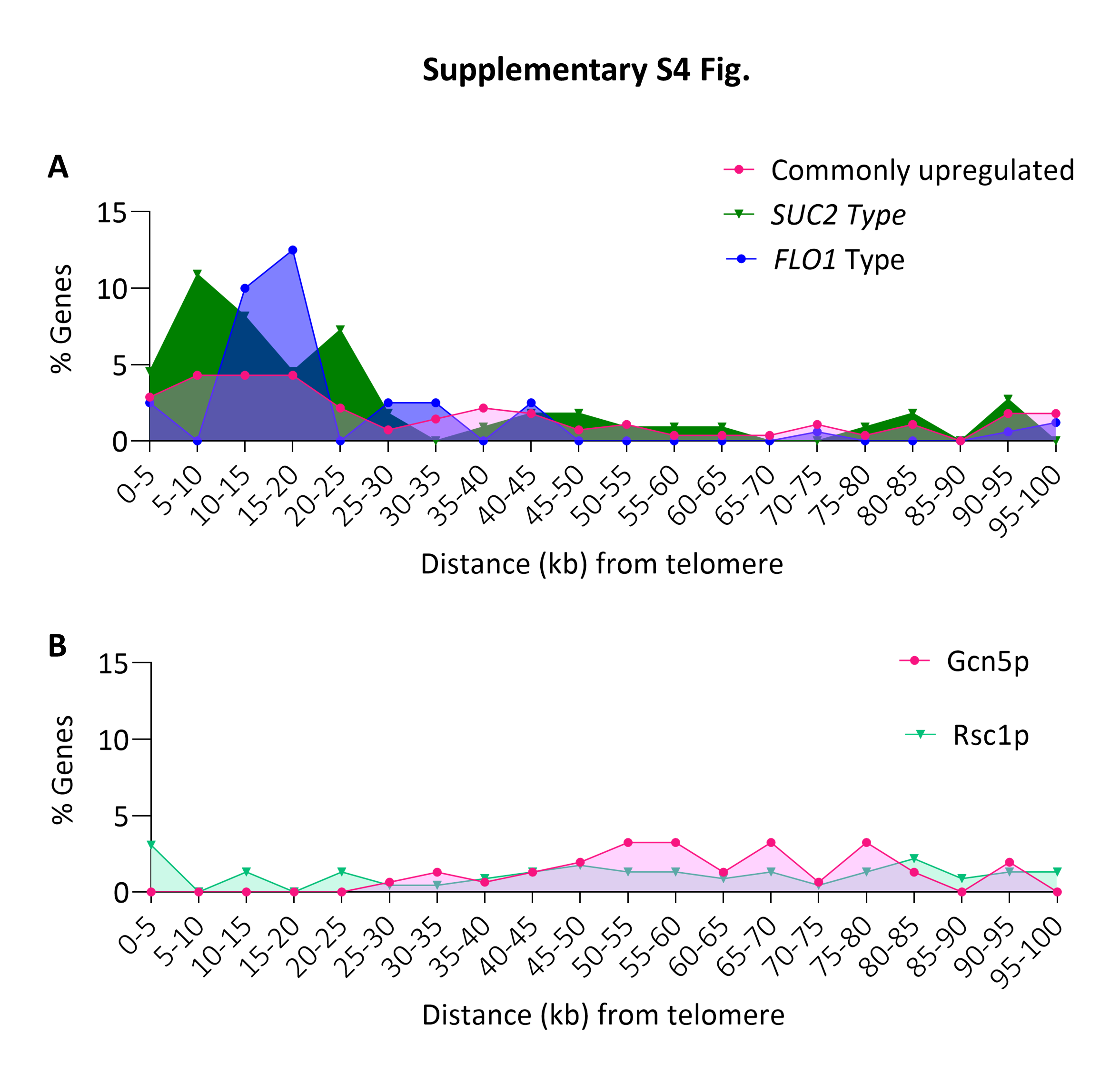

Supplement: S4 Fig — (A) Distribution of the 429 commonly upregulated genes over the first 100 kb regions from the telomeres divided into 5 kb regions and separated into FLO1-type (blue), SUC2-type (green), and the remaining commonly upregulated genes (pink). The percentage of genes in each group was calculated. (B) Distribution of Gcn5p and Rsc1p occupancy over the first 100 kb regions from the telomeres divided into 5 kb regions. Occupancy of each protein in each 5 kb region is shown as a % of total protein occupancy over this region. It is important to note that Gcn5p and Rsc1p do not co-localise with the sub-telomeric sites of enrichment of the FLO1- and SUC2-type genes, thus acting as negative controls to support the observation of the exclusive FLO1- and SUC2-type gene localization as being unique to Tup1p and Cyc8p regulated genes. Gcn5p and Rsc1p occupancy data were extracted from Rossi et al., 2021 [57]. (TIF) [file pgen.1010876.s007.TIF]

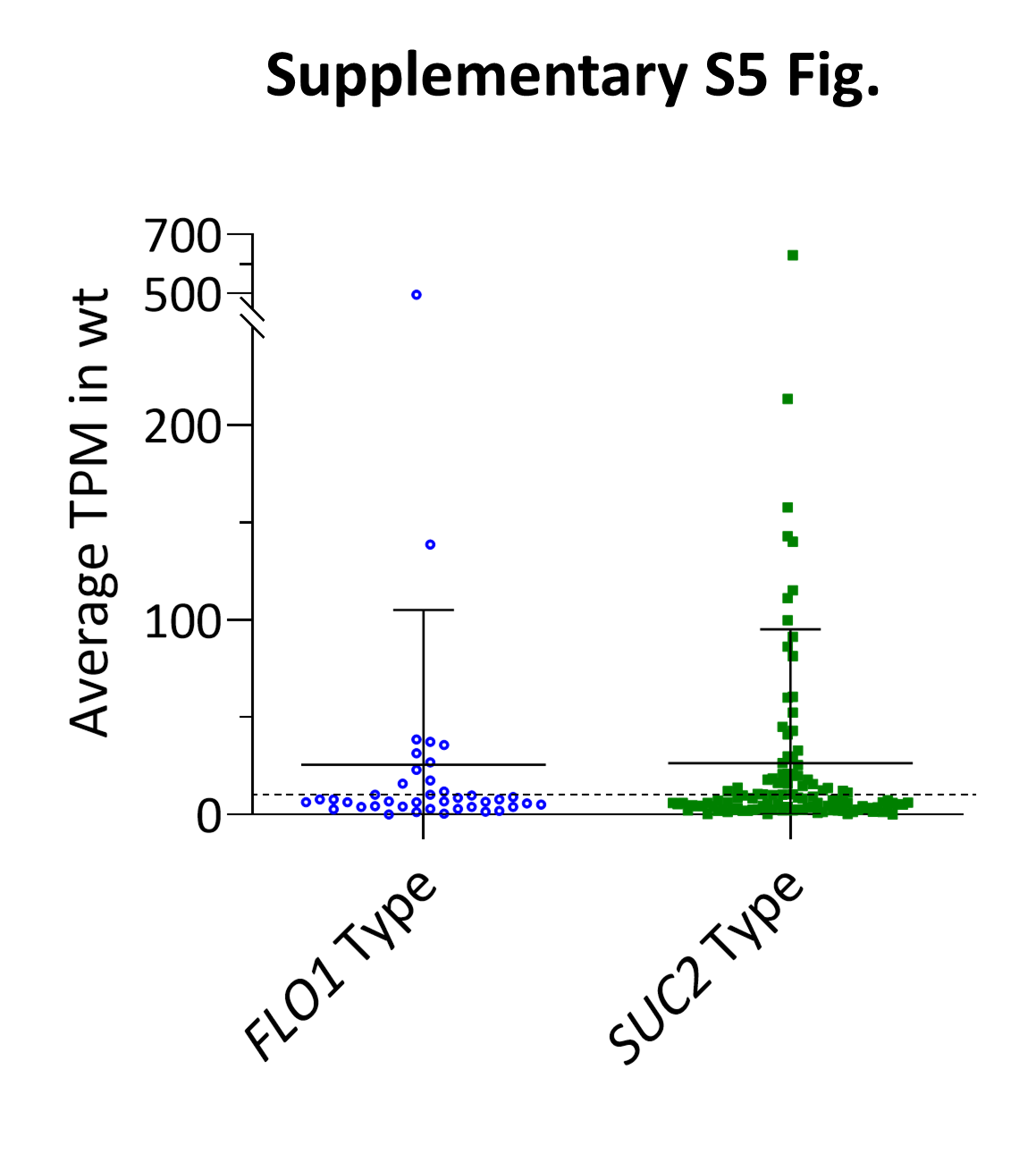

Supplement: S5 Fig — Scatterplot to show the average transcript per million (TPM) values from the three biological wt replicates for the FLO1- and SUC2-type genes. We assigned a cut-off of average TPM values ≤10 as ‘off’ in wt, represented by the dashed line. (TIF) [file pgen.1010876.s008.TIF]

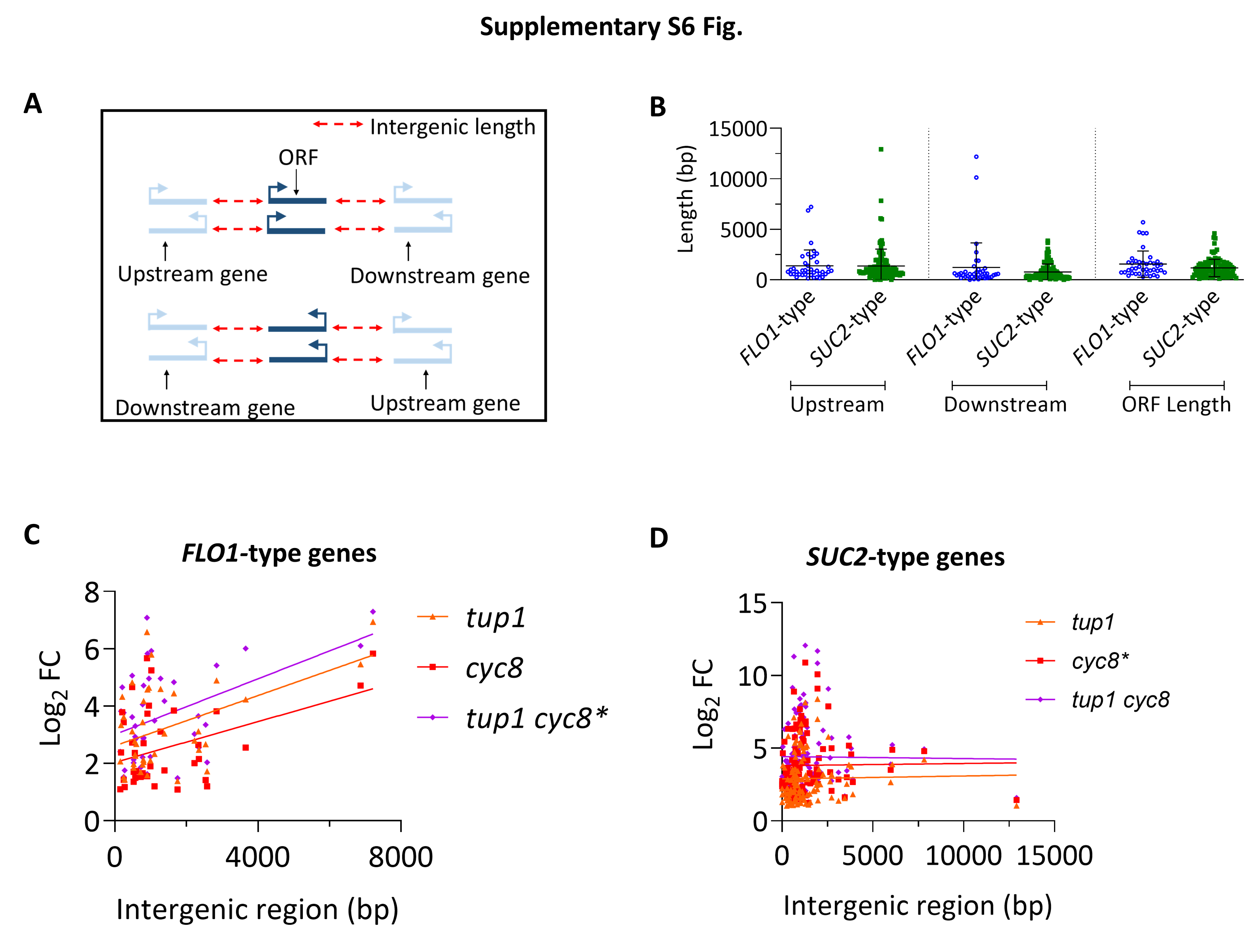

Supplement: S6 Fig — (A) Schematic depicting how intergenic length was calculated. (B) Scatterplot depicting the up- and downstream intergenic lengths of FLO1- and SUC2-type genes and the closest protein coding gene; also shown are the ORF lengths of FLO1- and SUC2-type genes (from SGD) (75). Correlating FLO1- and SUC2-type gene upstream intergenic region length with gene de-repression. Graphs depicting the length of the upstream intergenic length (X axis) and the change in transcription compared to wt (Y axis) for the (C) FLO1-type and (D) SUC2-type genes in tup1, cyc8 and tup1 cyc8 mutant strains. A line of best fit is shown for each strain. For FLO1-type genes (C), a two tailed Spearman correlation showed a statistically significant correlation between the upstream intergenic length and upregulation of transcription in the tup1 cyc8 strain compared to wt (P = 0.0294). For SUC2-type genes (D), a two tailed Spearman correlation showed a significant correlation in the cyc8 strain (p = 0.0449). (TIF) [file pgen.1010876.s009.TIF]

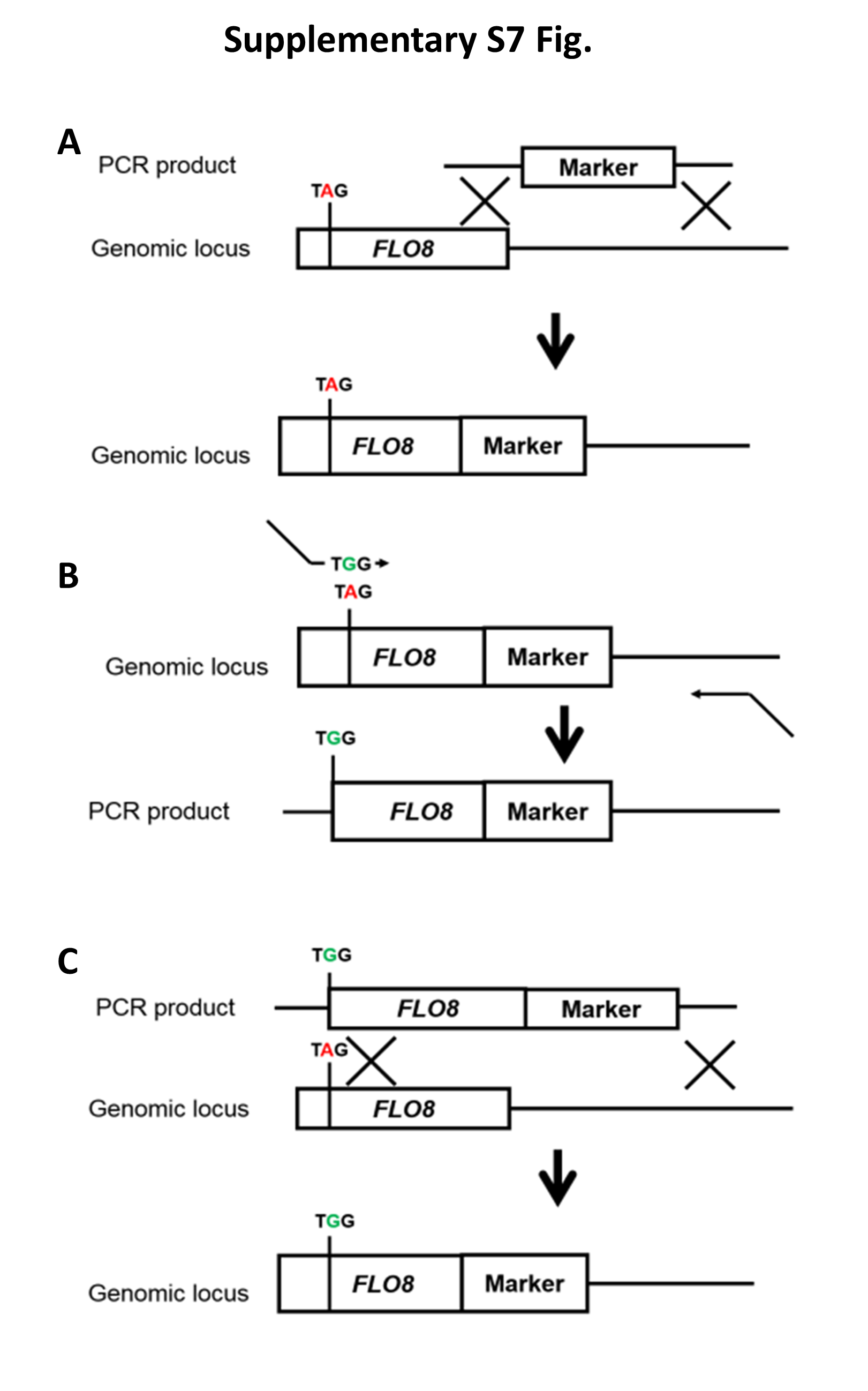

Supplement: S7 Fig — (A) In the first round of mutagenesis, a PCR product containing a selectable marker integrates into the position immediately downstream of the FLO8 3’ ORF by homologous recombination. The resulting strain contains a genomic copy of FLO8 immediately followed by the marker. (B) A forward primer was designed with homology to the FLO8 5’ ORF that contained an A-G base-pair substitution corresponding to position +425 of the FLO8 ORF. This was used in conjunction with a reverse primer with homology to an intergenic region downstream of FLO8. Using genomic DNA from the strain with the selectable marker directly downstream of the FLO8 ORF; these primers were used to generate a PCR product that contained the majority of the FLO8 ORF, but with the A-G point mutation at position +425. This product also contained the selectable marker adjacent to the FLO8 3’ ORF. (C) A second transformation was carried out in a wild type BY4741 strain using the PCR product containing the point mutation at position +425 in the FLO8 ORF. This resulted in a strain with a genomic copy of FLO8 containing a G at position +425 in place of an A. This strain also contained a selectable marker immediately downstream of the FLO8 ORF. The resulting strain (YMC19, FLO8+) was sequenced to confirm the point mutation. The restored FLO8 gene in YMC19 was subsequently tagged with a 9-Myc epitope to generate strain YMC34. (TIF) [file pgen.1010876.s010.TIF]

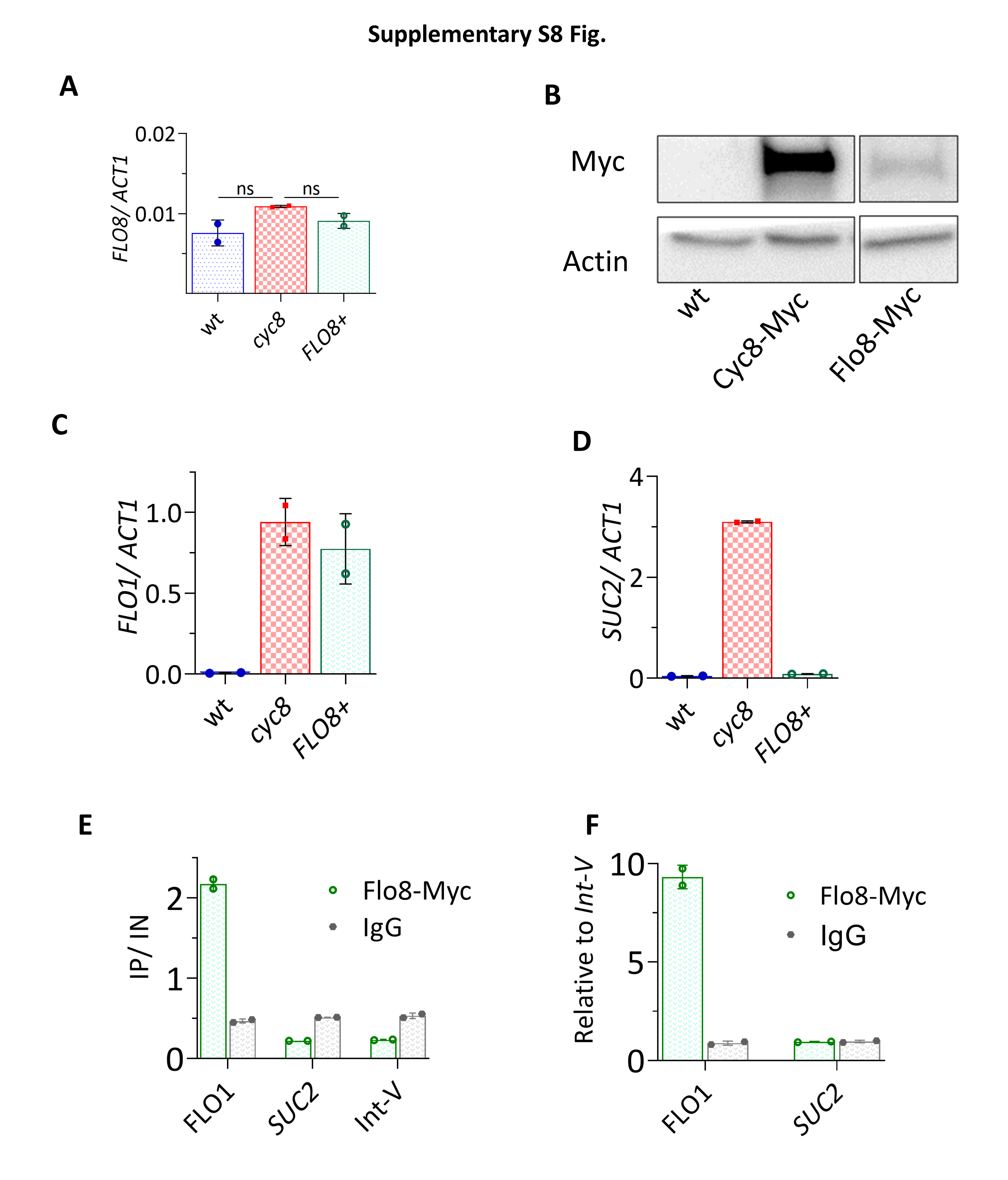

Supplement: S8 Fig — (A) Transcription from the non-functional FLO8 gene in wt and cyc8, and from the restored FLO8 gene in YMC19 (FLO8+), was analysed by RT-qPCR (error bars represent SD, n = 2). (B) Expression of Flo8-Myc in YMC34 was confirmed by western blot analysis. Wt and a Cyc8-myc strain were used as controls. Actin was used as a loading control. The proteins detected for Cyc8-Myc and Flo8-Myc were of the expected size. (C) FLO1 and (D) SUC2 mRNA levels detected by RT-qPCR in wt, cyc8 and the FLO8+ strain. The result shows that FLO1 is transcribed in the FLO8+ strain, whilst SUC2 is not transcribed (error bars represent SD, n = 2). The data in C is the same data shown in Fig 6G. (E) Flo8-Myc occupancy (IP/in) at the FLO1 promoter, SUC2 promoter, and at a negative control region, Int-V. (F) Flo8-Myc occupancy data from (E) shown as ‘relative occupancy’ following normalisation to Int-V (error bars represent SD, n = 2). The Flo8-Myc relative occupancy data shown here for FLO1 is the same data shown in Fig 6H. The results in C-F show that the impact of Flo8-Myc expression are specific for FLO1; Flo8-Myc does not occupy the SUC2 promoter, and SUC2 remains repressed in the FLO8+ strain. (TIF) [file pgen.1010876.s011.TIF]

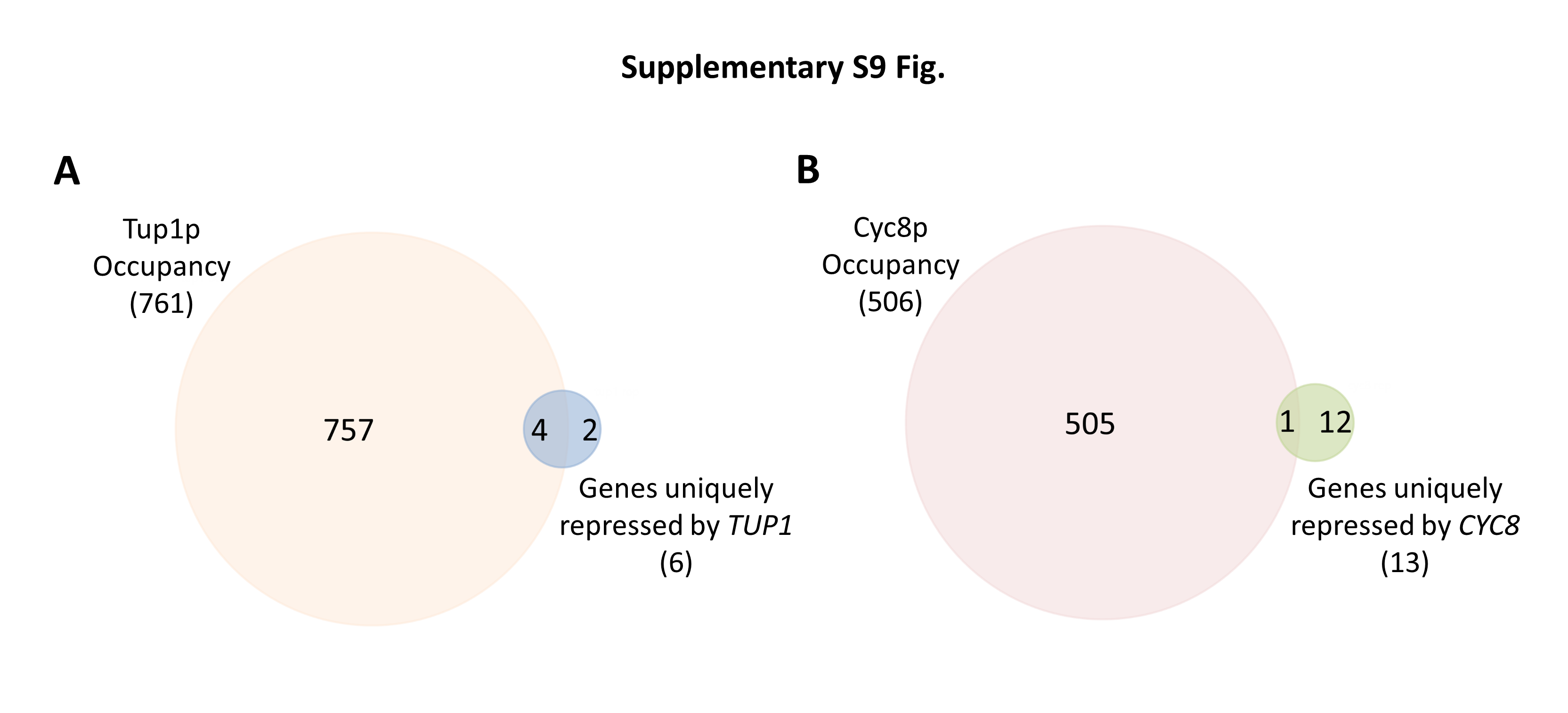

Supplement: S9 Fig — (A) Venn diagram showing the overlap between global Tup1p occupancy and genes identified as being uniquely repressed by TUP1 (see Fig 3F). (B) Venn diagram showing the overlap between global Cyc8p occupancy and genes identified as being uniquely repressed by CYC8 (see Fig 3D). Tup1p and Cyc8p occupancy data were retrieved from Rossi et al., 2021 [57]. (TIF) [file pgen.1010876.s012.TIF]

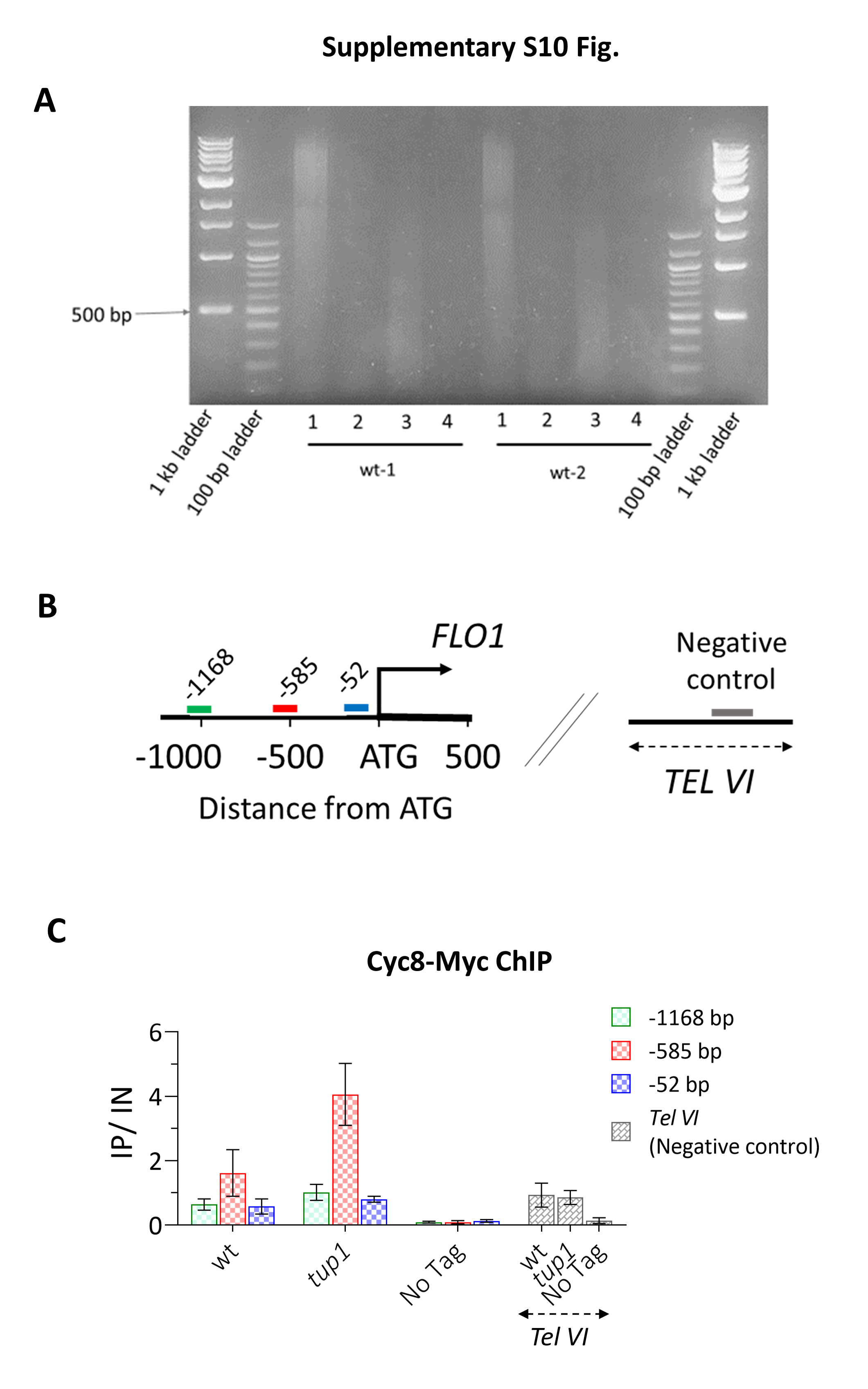

Supplement: S10 Fig — (A) Representative agarose gel to show DNA fragment size before and after chromatin sonication. A 1 kb DNA ladder and a 100 bp ladder (NEB) are indicated. The gel shows results from two wt samples (wt-1 and wt-2). For each sample the DNA included was 1: pre-sonicated input genomic DNA (RNase treated); 2: pre-sonicated input genomic DNA (RNase and DNase treated); 3: sonicated input DNA (RNase treated); 4: sonicated input DNA (RNase and DNase treated). (B) Schematic of the amplicons used for ChIP analysis across the FLO1 promoter and at the Tel-VI negative control region. (C) Cyc8-Myc occupancy across the FLO1 promoter region to show specific enrichment at -585 bp in wt and tup1. Cross linked chromatin from a Cyc8-Myc strain (wt), tup1/Cyc8-Myc strain (tup1), and an untagged strain (No tag) were immunoprecipitated with antibodies against the Myc tag. IP/IN for each of the indicated amplicons, as well as a negative control region within the right arm of Tel-VI, are shown. Mean and standard deviation are shown, * = p≤0.05, ** = p≤0.005 obtained from One-Way ANOVA analysis (n = 3–4). (TIF) [file pgen.1010876.s013.TIF]

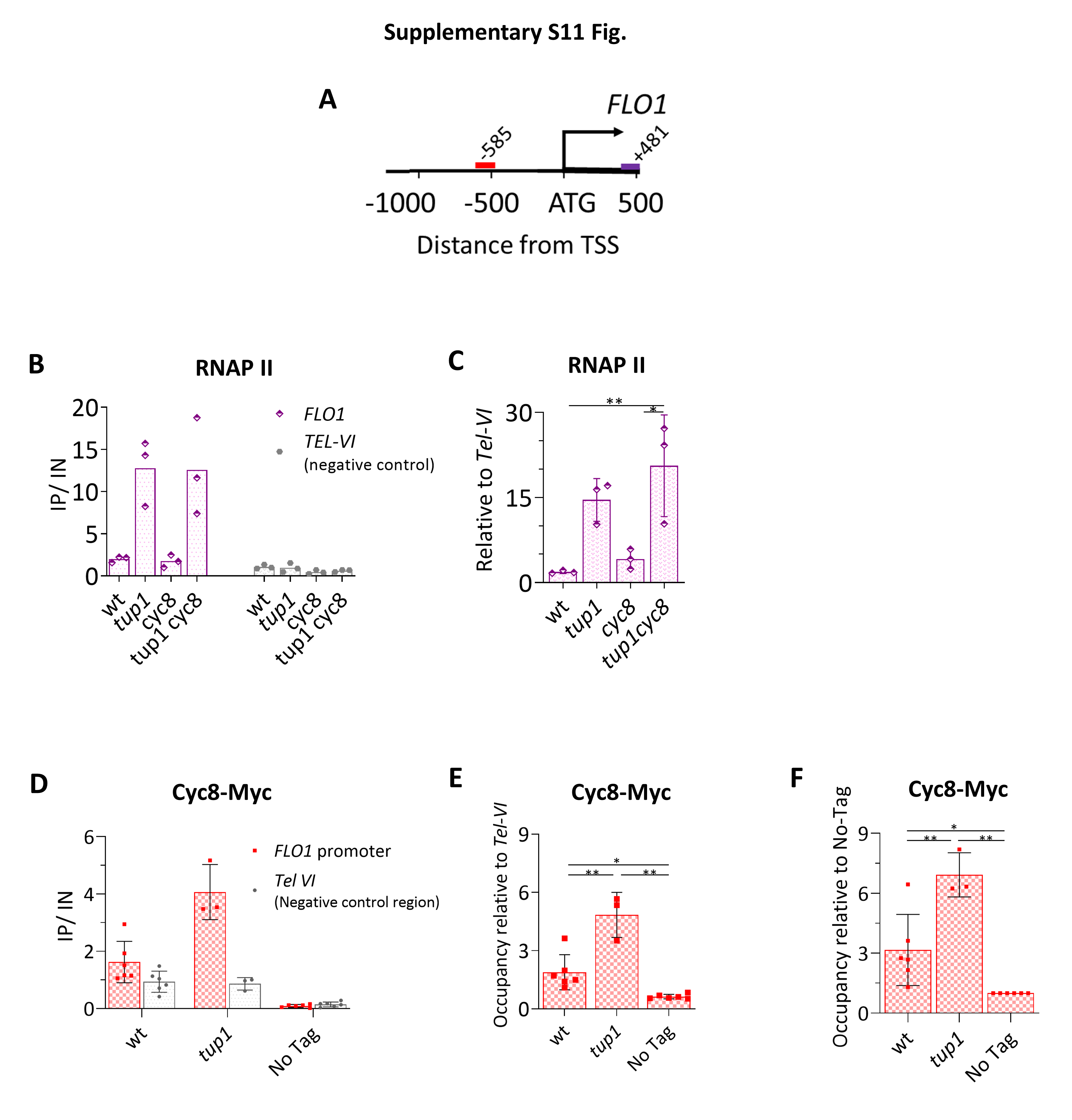

Supplement: S11 Fig — (A) Schematic to illustrate the amplicons used for ChIP analysis at the FLO1 promoter and ORF. (B-C) RNA polymerase II (RNAP II) occupancy at the FLO1 ORF in wt, tup1, cyc8 and tup1 cyc8 mutant strains. (B) IP/ IN for RNAP II occupancy at the FLO1 ORF and the negative control region (Tel-VI) are shown. (C) RNAP II occupancy at the FLO1 ORF normalised to Tel-VI to yield ‘relative occupancy’. (D-F) Cyc8-Myc occupancy at the FLO1 promoter. Chromatin from a Cyc8-Myc strain (wt), a tup1/Cyc8-Myc strain (tup1), and an untagged strain (No tag), were immunoprecipitated with antibodies against the Myc tag. (D) IP/IN for Myc occupancy at the FLO1 promoter and the negative control region (Tel-VI), are shown. (E) Cyc8-Myc occupancy at the FLO1 promoter normalized to occupancy at Tel-VI to yield ‘relative occupancy’. (F) Cyc8-Myc relative occupancy following normalization to relative occupancy in the No Tag strain. In all graphs mean and standard deviation are shown, * = p≤0.05, ** = p≤0.005 obtained from One-Way ANOVA analysis (n = 3–6). (TIF) [file pgen.1010876.s014.TIF]

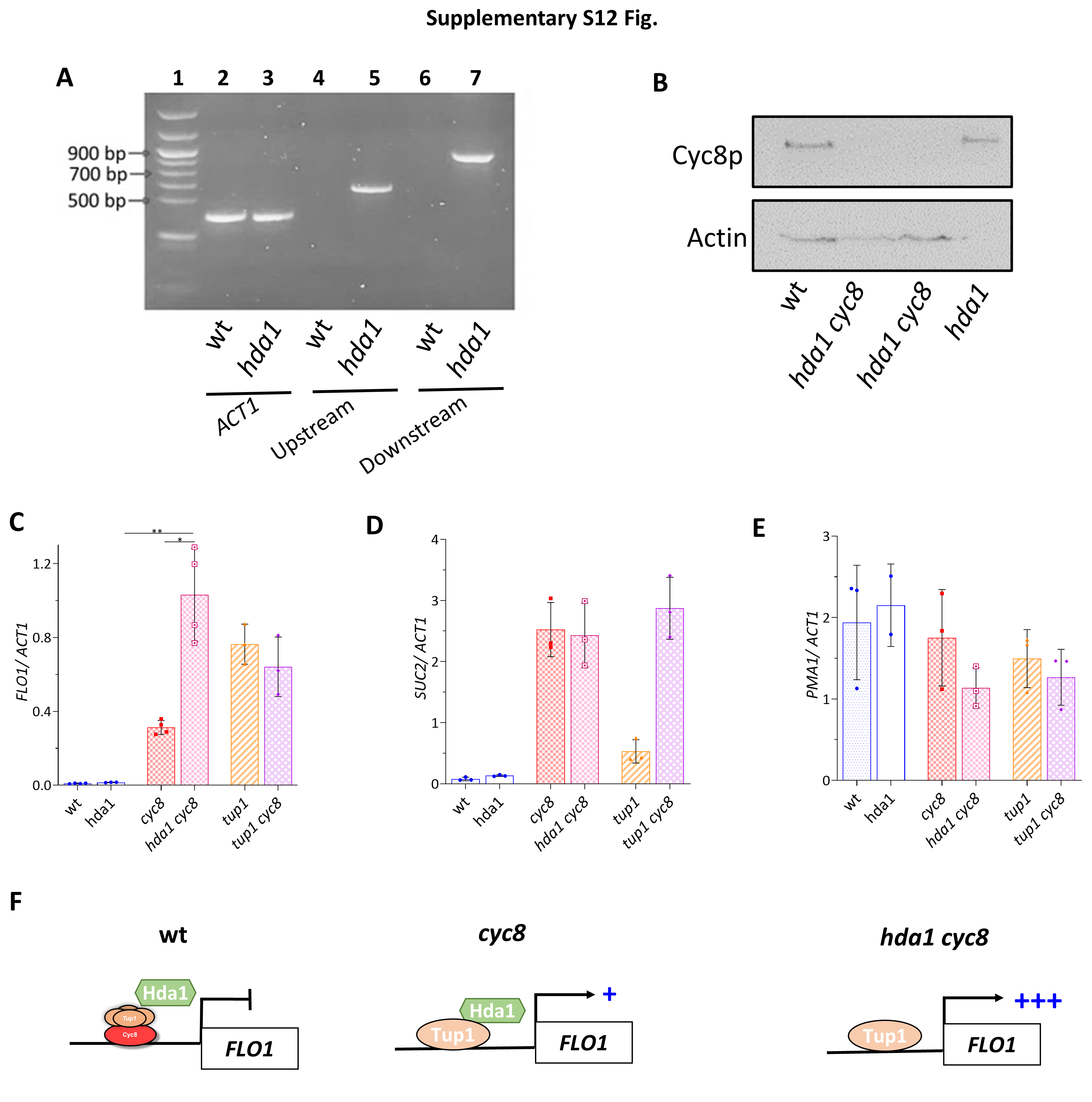

Supplement: S12 Fig — (A) PCR results from genomic DNA of the strains indicated to confirm the hda1::KAN mutant strain. Lane 1: 10 bp marker (NEB), lane 2, 3: PCR using primers for ACT1 (positive PCR control), lanes 4, 5: PCR using primers upstream of HDA1 (hda1DFconF) and internal to hda1::KAN (KanB), lanes 6, 7: PCR using primers downstream of HDA1 (hda1DFconR) and internal to hda1::KAN (KanC). (B) Western Blot analysis to confirm the deletion of CYC8 in a hda1 mutant strain (in duplicate). β-actin was used as a loading control. Bands detected were of the expected size. RT-qPCR analysis of transcription of (C) FLO1, (D) SUC2 and (E) PMA1 in the strains indicated. Values were normalised to ACT1 mRNA and error bars reflect standard deviation (n = 3–4). (RT-qPCR data of FLO1 and SUC2 in the wt, tup1, cyc8 and tup1 cyc8 strains has previously been shown in Fig 2). (F) Schematic to depict Tup1p-dependent role of Hda1p in FLO1 repression. In a cyc8 mutant strain we propose that Hda1p, in association with Tup1p, represses FLO1 in the absence of CYC8. Loss of both CYC8 and HDA1 results in high FLO1 de-repression (compare FLO1 mRNA levels in hda1 cyc8 and tup1 cyc8). This result is specific to FLO1. Transcription at SUC2 and PMA1 are not significantly affected. (TIF) [file pgen.1010876.s015.TIF]
