# Supplementary material for: Systematic analysis of tup1 and cyc8 mutants reveals distinct roles for TUP1 and CYC8 and offers new insight into the regulation of gene transcription by the yeast Tup1-Cyc8 complex
Source: PLoS Genet. 2023 Aug 11;19(8):e1010876. doi: 10.1371/journal.pgen.1010876 (PMC10446238; doi:10.1371/journal.pgen.1010876)
Supplement: S1 Table — (DOCX) [file pgen.1010876.s016.docx]

**S1 Table. Transcription factor DNA binding motifs enriched upstream of *FLO1*- and *SUC2*-type genes.**

| **Transcription**  **Factor** | **% Genes** | | **Documented Interaction** | | **Transcription Factor Function** |
| --- | --- | --- | --- | --- | --- |
|  | ***FLO1*-Type** | ***SUC2*-Type** | **Tup1p** | **Cyc8p** |  |
| **TF binding motifs found at more *FLO1*-type than *SUC2*-type genes** | | | | | |
| **Yap1** | 82.1 | 67.3 | G (1) | G (1) | Activates transcription of anti-oxidant genes in response to oxidative stress |
| **Hac1** | 74.4 | 61.8 | G (1) | P (1, 2) | Regulates unfolded protein response |
| **Gcn4** | 59 | 39.1 | G - Weak Interaction (3) | G -Strong Interaction (3) | Transcriptional activator of amino acid biosynthetic genes |
| **TF binding motifs found at more *SUC2*-type than *FLO1*-type genes** | | | | | |
| **Rtg1/ Rtg3** | 79.5 | 90.9 | No | P (4) | Rtg1p and Rtg3p form a complex. RTG3 = TF for retrograde (RTG) and TOR pathways; forms a complex with Rtg1p |
| **Msn2** | 66.6 | 83.6 | No | No | Stress-responsive transcriptional activator |
| **Msn2/Msn4/ Nrg1/ Rph1** | 61.5 | 74.6 | No | P (Nrg1) | Msn4 - Stress-responsive transcriptional activator. Rph1 - Histone demethylase |
| **Nrg1** | 46.2 | 72.7 | No (5) | P (5, 6) | Negative regulator of glucose-repressed genes |
| **Upc2** | 33.3 | 47.3 | No (7) | No (7) | Sterol regulatory element binding protein |
| **Hap1** | 25.6 | 41.8 | G (8) | G (2) | Regulation of gene expression in response to levels of heme and oxygen. Acts as activator or repressor |

**S1 Table. DNA binding motifs upstream of *FLO1*- and *SUC2*-type genes.** Information includes the name of the transcription factor (TF), the percentage of genes where the DNA binding motif appears at least once in the upstream region of *FLO1*- and *SUC2*-type genes (9). The known interaction between the TF and the subunits of the Tup1-Cyc8 complex, which may be either genetic (G) or physical (P). The known function of the TF, retrieved from the SGD website (10). Motifs that occur in less than 40% of genes in both cohorts of genes were not included for this analysis. The percentage of *FLO1-* or *SUC2-*type genes that had each motif was calculated. Shown here are only those motifs that show more than 10% difference in the proportion of genes containing that motif in each cohort.

**References**

1. Kumawat,R. and Tomar,R.S. (2022) Heavy metal exposure induces Yap1 and Hac1 mediated derepression of GSH1 and KAR2 by Tup1-Cyc8 complex. *J Hazard Mater*, **429**, 128367.

2. Kliewe,F., Engelhardt,M., Aref,R. and Schüller,H.-J. Promoter recruitment of corepressors Sin3 and Cyc8 by activator proteins of the yeast Saccharomyces cerevisiae.

3. Kim,S.J., Swanson,M.J., Qiu,H., Govind,C.K. and Hinnebusch,A.G. (2005) Activator Gcn4p and Cyc8p/Tup1p are interdependent for promoter occupancy at ARG1 in vivo. *Mol Cell Biol*, **25**, 11171–83.

4. Conlan,R.S., Gounalaki,N., Hatzis,P. and Tzamarias,D. (1999) The Tup1-Cyc8 Protein Complex Can Shift from a Transcriptional Co-repressor to a Transcriptional Co-activator*. *Journal of Biological Chemistry*, **274**, 205–210.

5. Lettow,J., Kliewe,F., Aref,R. and Schüller,H.-J. (2023) Functional characterization and comparative analysis of gene repression-mediating domains interacting with yeast pleiotropic corepressors Sin3, Cyc8 and Tup1. *Curr Genet*, 10.1007/s00294-023-01262-6.

6. Park,S.H., Koh,S.S., Chun,J.H., Hwang,H.J. and Kang,H.S. (1999) Nrg1 is a transcriptional repressor for glucose repression of STA1 gene expression in Saccharomyces cerevisiae. *Mol Cell Biol*, **19**, 2044–50.

7. Jordá,T. and Puig,S. (2020) Regulation of Ergosterol Biosynthesis in Saccharomyces cerevisiae. *Genes (Basel)*, **11**.

8. Zhang,L. and Guarente,L. (1994) Evidence that TUP1/SSN6 has a positive effect on the activity of the yeast activator HAP1. *Genetics*, **136**, 813–817.

9. Monteiro,P.T., Oliveira,J., Pais,P., Antunes,M., Palma,M., Cavalheiro,M., Galocha,M., Godinho,C.P., Martins,L.C., Bourbon,N., *et al.* (2020) YEASTRACT+: a portal for cross-species comparative genomics of transcription regulation in yeasts. *Nucleic Acids Res*, **48**, D642-d649.

10. Cherry,J.M., Adler,C., Ball,C., Chervitz,S.A., Dwight,S.S., Hester,E.T., Jia,Y., Juvik,G., Roe,T., Schroeder,M., *et al.* (1998) SGD: Saccharomyces Genome Database. *Nucleic Acids Res*, **26**, 73–9.
