# Supplementary material for: Systematic analysis of tup1 and cyc8 mutants reveals distinct roles for TUP1 and CYC8 and offers new insight into the regulation of gene transcription by the yeast Tup1-Cyc8 complex
Source: PLoS Genet. 2023 Aug 11;19(8):e1010876. doi: 10.1371/journal.pgen.1010876 (PMC10446238; doi:10.1371/journal.pgen.1010876)
Supplement: S2 Table — (DOCX) [file pgen.1010876.s017.docx]

**S2 Table. Yeast strains used in this study.**

| **Strain** | **Genotype** | **Description** | **Source** |
| --- | --- | --- | --- |
| BY4741 | *Mat*a *his3Δ1* *leu2Δ0* *met15Δ0* *ura3Δ0* | wt, Fig 1-3,6A, 6B, 6D, 6E, 6G, 6I. Labelled as ‘No tag’ in Fig 6C & 6F.  S Figs 1-3, 8A-D, 10A, 11B, 11C, 12A-E.  Labelled as No Tag in 10C, 11D-F | (Brachmann et al., 1998) (1) |
| YAFTCD5 | *Mat*a*; his3Δ1; leu2Δ0; met15Δ0; ura3Δ0;* *tup1Δ::KanMX4* | *tup1* Fig 1-5, 6A, 6B, 6D, 6E, 6I.  S Figs 1-3, 6C, 6D, 11B, 11C, 12C-E | ResGen library |
| YAFTCD4 | *Mat*a; *his3Δ1; leu2Δ0; met15Δ0; ura3Δ0*; *cyc8Δ::KanMX4* | *cyc8* Figs 1-5, 6A, 6B, 6D, 6E, 6G  S Figs 1-3, 6C, 6D, 8A, 8C, 8D, 11B, 11C, 12C-E | ResGen library |
| YMC12 | *Mat*a*; his3Δ1; leu2Δ0; met15Δ0; ura3Δ0; tup1Δ::KanMX4; cyc8Δ::URA3* | *tup1 cyc8,* Figs1-6  S Figs 1-3, 6C, 6D, 11B, 11C, 12C-E | Fleming lab |
| YPOD1 | *Mat*a*; his3Δ1; leu2Δ0; met15Δ0; ura3Δ0;*  *CYC8-9Myc::KanMX4* | Cyc8-Myc, labelled as wt in Figs 6C & 6F  S Figs 10C, 11D-F | Fleming lab |
| YMC11 | *Mat*a*; his3Δ1; leu2Δ0; met15Δ0; ura3Δ0;*  *CYC8-9Myc::KanMX4; tup1::URA3* | Cyc8-Myc, *tup1*. Figs 6C & 6F.  S Figs 10C, 11D-F. | Fleming lab |
| YMC19 | *BY4741* (*Mat*a *his3Δ1* *leu2Δ0* *met15Δ0* *ura3Δ0) FLO8A245G::hph-NT1* | *FLO8+* untagged strain  Figs 6G & 6I.  S Figs 8A, 8C, 8D | This study |
| YMC34 | BY4741 (*Mat*a *his3Δ1* *leu2Δ0* *met15Δ0* *ura3Δ0) FLO8A245G::9myc::KanMX* | *FLO8+* tagged strain, Fig 6H.  S Figs 8E & 8F | This study |
| YAFTCD2 | *Mat*a; *his3Δ1; leu2Δ0; met15Δ0; ura3Δ0*; *hda1Δ::KanMX4* | *hda1,* Discussion,  S Figs 12A-E | ResGen library |
| YBL19 | *Mat*a; *his3Δ1; leu2Δ0; met15Δ0; ura3Δ0*; *hda1Δ::KanMX4 cyc8Δ::HIS3* | *hda1 cyc8,* Discussion,  S Figs 12B-E | This study |

**References**

1. Brachmann CB, Davies A, Cost GJ, Caputo E, Li J, Hieter P, et al. Designer deletion strains derived from Saccharomyces cerevisiae S288C: a useful set of strains and plasmids for PCR-mediated gene disruption and other applications. Yeast. 1998 Jan 30;14(2):115–32.
