# Supplementary material for: Systematic analysis of tup1 and cyc8 mutants reveals distinct roles for TUP1 and CYC8 and offers new insight into the regulation of gene transcription by the yeast Tup1-Cyc8 complex
Source: PLoS Genet. 2023 Aug 11;19(8):e1010876. doi: 10.1371/journal.pgen.1010876 (PMC10446238; doi:10.1371/journal.pgen.1010876)
Supplement: S3 Table — (DOCX) [file pgen.1010876.s018.docx]

**S3 Table. Antibodies used in Western immunoblotting.**

| **Protein** | **Dilution** | **Source** |
| --- | --- | --- |
| β-actin | 1:3000 | Abcam (ab8224) |
| Myc | 1:5000 | Millipore (05-724) |
| Cyc8 | 1:500 | Santa Cruz (sc-11953) |
| Tup1 | 1:5000 | J. Reese |

Protein lysates (30μg) were electrophoresed on 12% acrylamide gels, and protein was transferred to a PVDF Immobilon membrane (Millipore). All antibodies were diluted in 5 % skimmed milk in Tris-buffered saline with 0.05% Tween 20 (TBST). Blots were developed with ECL Western Blotting Substrate (Pierce).
