# Supplementary material for: Systematic analysis of tup1 and cyc8 mutants reveals distinct roles for TUP1 and CYC8 and offers new insight into the regulation of gene transcription by the yeast Tup1-Cyc8 complex
Source: PLoS Genet. 2023 Aug 11;19(8):e1010876. doi: 10.1371/journal.pgen.1010876 (PMC10446238; doi:10.1371/journal.pgen.1010876)
Supplement: S4 Table — (DOCX) [file pgen.1010876.s019.docx]

**S4 Table. Oligonucleotides used in study.**

| **Name:** | **Sequence (5’-3’):** | **Description:** | **Distance relative to ATG:** |
| --- | --- | --- | --- |
| ***ACT1* ORF-F** | GAGGTTGCTGCTTTGGTTATTGA | *ACT1* transcription | +318 |
| ***ACT1* ORF-R** | ACCGGCTTTACACATACCAGAAC |  |  |
| ***FLO1* RT-F** | TACCACCACAGACGGGTTCT | *FLO1 t*ranscription/ ORF ChIP | +481 |
| ***FLO1* RT-R** | CAACAGTTGAACGCGGTTGC |  |  |
| ***FLO5* RT5'-F2** | GGATGGAAGTCTCCCTGACA | *FLO5* transcription | +635 |
| ***FLO5* RT5'-R2** | GGAAACGGCATTGGAGTAAA |  |  |
| ***FLO9* RT5'-F** | TCGTCACATTGCTGGGATTA | *FLO9* transcription | +105 |
| ***FLO9* RT5'-R** | TGCTGCATTCGAATATGTGG |  |  |
| ***FLO10* RT-F** | GCGGTTAGTTCTGACATCGAAAAT | *FLO10* transcription | +3164 |
| ***FLO10* RT-R** | TTTTGTCTCAGCAGCCTCTGAA |  |  |
| ***SUC2* RT486-F** | AGCTGCCAACTCCACTCAAT | *SUC2* transcription/  ORF ChIP | +486 |
| ***SUC2* RT486-R** | ATTTGGCAGCCGTCATAATC |  |  |
| ***SUR2*_F** | CAAGGCCCCTGCCATTAAT | *SUR2* transcription | +114 |
| ***SUR2*_R** | CACTCATTTCCGGCAGCAA |  |  |
| ***PHO3*_F** | CGTGAGTTTTTAGCGCAATATGG | *PHO3* transcription | +479 |
| ***PHO3*_R** | GCGAAAATTGGGAAACTGGTT |  |  |
| ***RNR3*_F** | ACTTTGAACTCGGCCATCGT | *RNR3* transcription | +424 |
| ***RNR3*_R** | CGAACGCTCCAGTGTCTTGA |  |  |
| ***TEL VI*-R 121 F** | CGTGTGTAGTGATCCGAACTCAGT | Control region (ChIP) | N/A |
| ***TEL VI*-R 121 R** | GACCCAGTCCTCATTTCCATCAATAG |  |  |
| ***INT-V*** | TAAGAGGTGATGGTGATAGGCGT | Control region (ChIP) | N/A |
| ***INT-V*** | CCCTCGGGTCAAACACTACAC |  |  |
| **NUC2-F** | CGC ATT TTT ATT ACT CTG AAC AGG | *SUC2* ChIP (Promoter) | -445 |
| **NUC2-R** | GGA CGT GGG GTC GAT TAA C |  |  |
| **NUC4-F (TATA)** | TGGAAGAAAGATTTGACGACTTT | *SUC2* ChIP (Promoter) | -168 |
| **NUC4-R (TATA)** | TGTTTCTTTTCAGGAGGAAGGA |  |  |
| **IPFLO1-F** | AAAGGAACATATTTCACTCTTGCTC | *FLO1* ChIP (Promoter) | -52 |
| **IPFLO1-R** | TCTGTTTACTGGTGACAAGAATTAAAA |  |  |
| **IPFLO3-F** | GCTTCCAGTATGCTTTCACG | *FLO1* ChIP (Promoter) | -585 |
| **IPFLO3-R** | GCCTACGTATTCTCCGTCAC |  |  |
| **IPFLO5-F** | TTGAATGGCACTAGTCGATCG | *FLO1* ChIP (Promoter) | -1168 |
| **IPFLO5-R** | TTAAACTTACGGCATCTTGAACATT |  |  |
| **CYC8_PRS_F** | GCAGCAGTTCCTCAGCAGCCACTCGACCCATTAACACAATAGATTGTACTGAGAGTGCAC | *CYC8* deletion | N/A |
| **CYC8_PRS_R** | AACAGAAGCTGCTTTGGTAGCTTCTTCAGCAGGACTAGCTGCTGTGCGGTATTTCACACCG |  |  |
| ***ACT1* F** | CTGAATTAACAATGGATTCTGG | PCR control | N/A |
| ***ACT1* R** | AGATACCTCTCTTGGATTGAGC |  |  |
| **Kan B-F** | CTGCAGCGAGGAGCCGTAAT | Strain confirmation (PCR) | N/A |
| **Kan C-R** | TGATTTTGATGACGAGCGTAAT |  |  |
| ***HDA1* DFcon-F** | TTGCAGCCTCCTTCTCAAT | Strain confirmation (PCR) | N/A |
| ***HDA1* DFcon-R** | CTCCTCCAAAACCACTGCTC |  |  |
| ***FLO8*mut*-F*** | GGCTTTTTGTATGAATGGTGGCAAAT | *FLO8* restoration | N/A |
| ***FLO8*DF-R** | GGTTCAGTTCACAGGGCTTA |  |  |
| ***FLO8*tag-F** | TACAAATGAAAATGATTTCAATTTTATTAATTGGGAAGGCCGTACGCTGCAGGTCGAC | *FLO8 tagging* | N/A |
| ***FLO8*pFA6-R** | AAGAGTTTTTATTTTTTATTATAATACTCAACACGTGACTATCGATGAATTCGAGCTCG |  |  |
