# Supplementary material for: Systematic analysis of tup1 and cyc8 mutants reveals distinct roles for TUP1 and CYC8 and offers new insight into the regulation of gene transcription by the yeast Tup1-Cyc8 complex
Source: PLoS Genet. 2023 Aug 11;19(8):e1010876. doi: 10.1371/journal.pgen.1010876 (PMC10446238; doi:10.1371/journal.pgen.1010876)
Supplement: S5 Table — (DOCX) [file pgen.1010876.s020.docx]

**S5 Table: Antibodies and conditions used for chromatin immunoprecipitation (ChIP)**

| **Antibody** | **Amount of antibody (μl)** | **Number of washes** | **Protein A or G** | **Source of antibody** |
| --- | --- | --- | --- | --- |
| RNA Pol II | 4.5 | 2 | A/G mix | Covance (MMS-126R) |
| Tup1 | 1.5 | 2 | A | J. Reese |
| Myc | 2.5 | 2 | G | Millipore (05-724) |

Antibodies were added to cell lysates and incubated with rotation at 4°C overnight. The anti-Tup1 antibody was a generous gift from J. Reese. The chromatin-antibody-bead complexes were collected by the addition of 30 µl of the appropriate Dynabead or Dynabeads mix and incubation for 2 hours at 4°C. All chromatin-antibody-bead complexes were washed in 1 ml FA lysis buffer for 5 minutes, followed by either one or two washes (as indicated in Table S4) in 1 ml ChIP wash buffer #1 (50 mM HEPES [pH 7.5], 0.5 M NaCl, 1 mM EDTA, 1 % Triton X-100, 0.1 % Sodium deoxycholate), either one or two washes (as indicated in Table S4) in 1 ml ChIP wash buffer #2 (10 mM Tris-Cl [pH 8.0], 0.25 M LiCl, 1 mM EDTA, 0.5 % NP-40, 0.5 % Sodium deoxycholate) and a single wash in 1 ml TE (pH 7.5). Beads were then resuspended in 250 µl ChIP elution buffer (25 mM Tris Cl [pH 7.5], 5 mM EDTA, 0.5 % SDS) and the chromatin was eluted after the sequential incubation at 65°C for 20 minutes, rotation for 10 minutes at room temperature and centrifugation at 16,363 rcf for 1 minute. The resultant supernatant was protease treated and the cross-links were reversed. Input (in) and immunoprecipitated DNA (IP) were purified using a QiaQuick PCR purification kit (Qiagen) according to manufacturer’s instructions.

DNAs were analysed in triplicate by real-time quantitative PCR (qPCR) using a SYBR Green Master Mix (ABI) and ABI Step-One Plus PCR machine.
